# Integrated remote sensing and aeromagnetic datasets for mapping iron mineralization potential in the El-Bahariya depression in the Western Desert of Egypt

**DOI:** 10.1038/s41598-026-59828-6

**Published:** 2026-06-25

**Authors:** Saif M. Abo Khashaba, Safaa M. Hassan, Mohamed F. Sadek, Noureldin Laban, Safwat S. Gabr, Mohamed R. Metwalli, Mabrouk Sami, Suhail S. Alhejji, Ioan V. Sanislav, Mostafa Nagy

**Affiliations:** 1 Geology Department, Faculty of Science, Kafrelsheikh University, Kafrelsheikh, 33516 Egypt; 2 National Authority for Remote Sensing and Space Sciences (NARSS), Cairo, 1564 Egypt; 3 Geosciences Department, College of Science, United Arab Emirates University, Al Ain, 15551 United Arab Emirates; 4 Geology and Geophysics Department, College of Science, King Saud University, P.O. Box 16, Riyadh, 2455, 11451 Saudi Arabia; 5 Economic Geology Research Centre (EGRU), College of Science and Engineering, James Cook University, Townsville, 4814 Australia

**Keywords:** Remote sensing, Aeromagnetic, Iron exploration, El-Bahariya, Hyperspectral PRISMA, Environmental sciences, Solid Earth sciences

## Abstract

The El-Bahariya depression in the Western Desert of Egypt is well-known for its iron ore deposits, with mineralization recorded in five well-known locations. This study is the first integration of hyperspectral PRISMA and aeromagnetic analysis for the Bahariya iron ore deposits. Automated lithological mapping of the study area was performed using machine learning algorithms (MLA) such as Random Forest (RF) and Support Vector Machine (SVM), applied to stacked (ASTER + Sentinel-2) data, achieving an overall accuracy of up to 91.73%, Kappa accuracy of 89.99%, and F1-score of 93.98%. Hyperspectral PRISMA data analysis identified diagnostic absorption features (1.95–2.3 μm) associated with hematite, goethite, and limonite. Advanced spectral methods, including Pixel Purity Index (PPI) and Spectral Angle Mapper (SAM), successfully discriminated between hematite and limonite concentrations, as validated by field surveys, an ASD spectroradiometer, and USGS laboratory spectra. Results revealed ferruginous sandstone distributions, iron-rich zones, and previously undetected high-potential mineralization targets. Complementary high-resolution aeromagnetic data, processed using edge detection filters and CET techniques (CET-GA, CET-PA), resolved structural controls on mineralization. Major NE-SW and NW-SE trending lineaments, alongside minor N-S and E-W structures, were identified as fluid conduits for hydrothermal iron oxide emplacement. Euler deconvolution constrained magnetic sources to shallow depths (< 2 km), aligning with surface-derived anomalies. High magnetic susceptibility zones correlated strongly with remote sensing-identified iron-rich areas, including known mines, and highlighted unexplored anomalies in the northwestern and central regions. Geochemically, we identified economic ore-grade ironstones distinct (FeO^t^ = 26–46 wt%, MnO < 0.11 wt%, P₂O₅ 0.45–0.84 wt%) from non-economic Mn-rich carbonate lenses (MnO 4.85–7.76 wt%) and barren siliceous rocks. The integration of spectral and magnetic datasets confirmed the spatial coherence of mineralization signals, along with the detection of new high-potential zones for iron mineralization, demonstrating the synergistic utility of these datasets in defining exploration targets. The proposed methods were highly effective in mapping iron oxide deposits within the four major zones: Nasser, Gabal-Ghurabi, El-Gadidah, and El-Harrah.

## Introduction

**Fig. 1:**
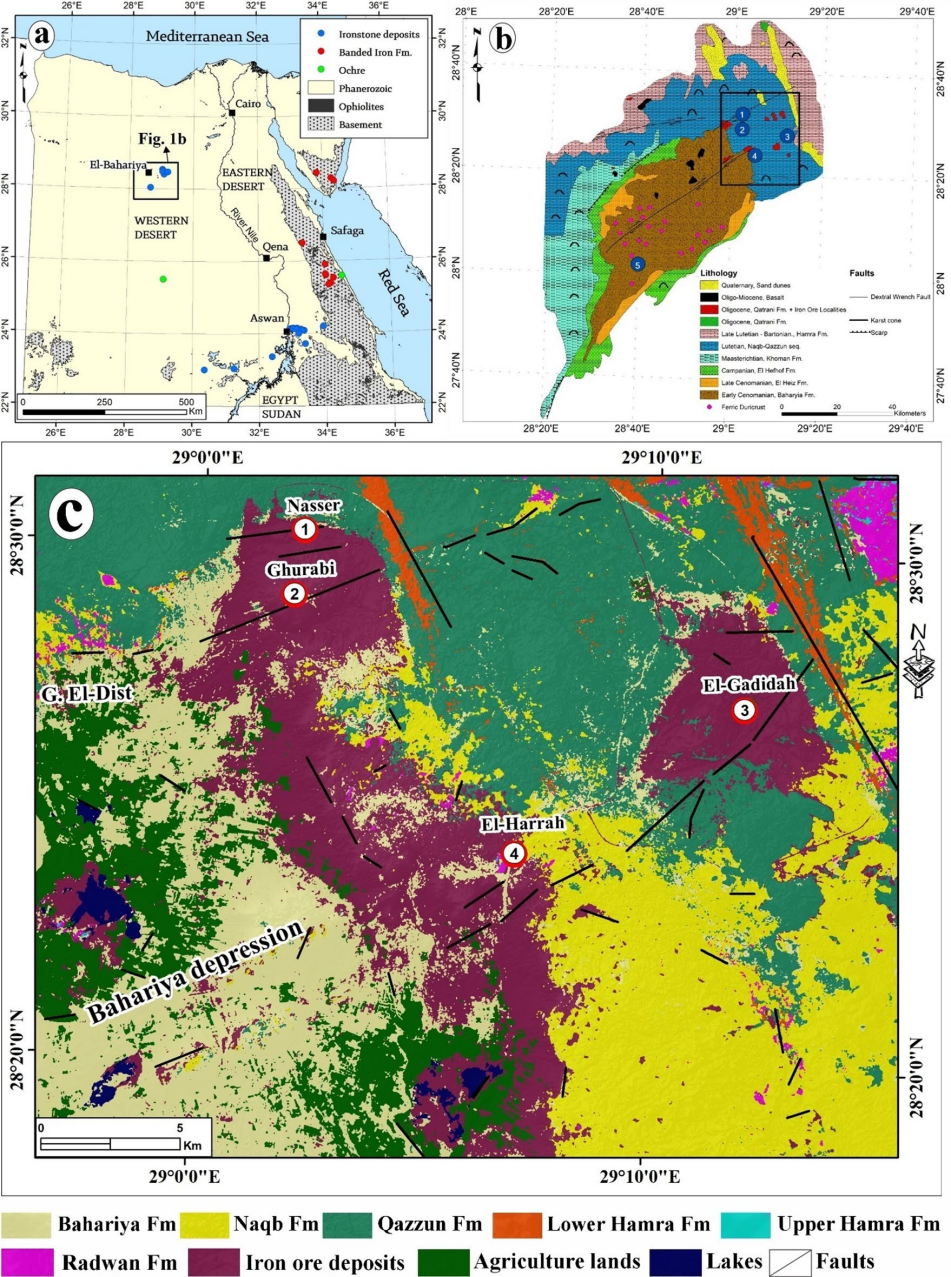
**(a)** A map showing the iron ore deposits of Egypt and the location of our study area [after 1]. The rectangle represents the location of the study area. (**b**) A geologic map of the El-Bahariya area showing the study area (black box), the locations of the five main ironstone deposits [(1) Nasser, (2) Gabal Ghurabi, (3) El-Gadidah, (4) El-Harrah, and (5) Al-Hayz] in the area. (**c**) Geological map of the El-Bahariya area based on the present work (integrated machine learning algorithms (stacked (ASTER + Sentinel-2-based RF-MNF), field observation, and geochemistry), previous works^35^. The figure was created by ArcGIS Desktop 10.8. (https://www.esri.com/enus/arcgis/products/arcgis- 148 desktop/overview/).

Exploration for metallic ore deposits has been well known in Egypt since the predynastic period (5500 to 3100 BC). Among all the metallic deposits in Egypt, hematite and limonite were the least exploited elements, followed by manganese and chromite, which were mined on a small scale, and then all the remaining metallic deposits^1^. The iron ore deposits of Egypt are sedimentary in nature and have been recorded in three main forms, namely, ironstone deposit, banded iron formation (BIF), or ochre (Fig. 1). They are located in several locations, which are concentrated mainly in Sinai and the Eastern and Western Deserts^2,3^.

The El-Bahariya area in the Western Desert of Egypt is well known for its ironstone deposits (Fig. 1), a term referring to iron deposits formed within Phanerozoic sediments. Five ironstone deposits have been recorded in the El-Bahariya area, namely (1) Nasser, (2) Gabal Ghurabi, (3) El-Gadidah, (4) El-Harrah, and (5) Al-Hayz (Fig. 1). The ore grade of the El-Gadidah area is considered to be the highest when compared to the other locations with lower manganese (Mn) content^1^. In 1972, the iron ore reserves of the El-Gadidah area were estimated to be approximately 135 Mt, directly prior to the initial exploitation work in the area^1^, which was considered the only commercial oolitic ironstone in northern Africa and southern Europe^4^. Recent calculations estimated the remaining mineable reserves in these areas to be approximately 63 million tonnes (Mt), which is sufficient for exploitation over the next 15–20 years at a rate of around 3–3.5 million tonnes per year (Mt/yr)^1^. The iron ore from El-Gadidah deposits is considered the primary iron raw material supplied to the Egyptian Iron and Steel Company, Helwan, Cairo, since 1973. Therefore, exploration for additional iron ore deposits is crucial for the continuation of the iron industry in Egypt, particularly those with low contamination.

Remote sensing applications are commonly used in lithological as well as different mineral exploration activities [e.g., 5–11]. Machine learning algorithms (MLAs), such as Random Forest (RF) and Support Vector Machines (SVM), are effectively utilized for precise automatic lithological mapping. They can operate on various remote sensing datasets, including hyperspectral data (e.g., PRISMA) and multispectral data (e.g., Landsat OLI, Sentinel-2, and ASTER) [e.g., 10, 11, 12]. Data from multispectral (ASTER, Landsat-8, Sentinel-2) and hyperspectral (PRISMA) remote sensing sensors have been widely used to map iron-rich deposits in different locations around the globe, e.g., Saudi Arabia, Canada, Sudan, Australia, Algeria, and Iran^13–15^. Hyperspectral sensors, such as PRISMA, capture reflectance spectra across hundreds of contiguous, narrow wavelength bands (400–2500 nm), enabling pixel-by-pixel identification of diagnostic absorption features characteristic of ore-bearing minerals. Unlike multispectral satellites (Landsat, Sentinel-2, ASTER), which are limited to 2–6 bands in the shortwave infrared (SWIR) region, PRISMA’s continuous spectral coverage in the VNIR and SWIR bands resolves subtle mineralogical variations that distinguish economic ore mineralogy from barren country rock^11,12^. When combined with field spectroscopy (ASD FieldSpec) and synthetic aperture radar, such as Sentinel-1, hyperspectral remote sensing provides a comprehensive, multi-scale framework for mineral exploration across diverse deposit types, including rare earth elements, lithium pegmatites, gold systems, and porphyry copper deposits in structurally controlled geotectonic settings worldwide^10,11^.

Aeromagnetic data has proved its effectiveness in identifying hydrothermal alteration zones linked to mineral deposits. By analyzing this data, it is possible to map structural features that act as pathways for hydrothermal alteration or reveal altered areas associated with porphyry minerals^16–18^. Aeromagnetic data offer several key benefits compared to other geophysical techniques, particularly their ability to cover large areas quickly and cost-effectively^19^.

The exploration of mineral deposits is a worldwide phenomenon, with numerous researchers employing various approaches and techniques in different regions to achieve their objectives. The nature of the deposits typically determines which geophysical methods are used; for example, Guo et al.^20^ found that in two separate areas of Gansu Province, China, the ground magnetic method successfully identified gold mineralization linked to host sediments and sulfides, such as pyrrhotite. However, at a third site within the same region, the magnetic technique was unable to detect mineralization due to interference from the response of the igneous host rock, which masked the magnetic signal. Accordingly, a researcher must thoroughly understand the area’s geology to select the most appropriate geophysical approach for the study. Various integrated remote sensing approaches have been commonly applied to explore ore deposits^21^. Ogah and Abubakar^22^ employed a combination of magnetic and radiometric data to identify potential zones of mineralization in northwestern Nigeria.

Much of the mineralization occurrences are understood to be controlled by structural elements. Consequently, when analyzing magnetic data, it is crucial to closely examine highlighted structures, hydrothermal alterations, and any concealed intrusive bodies that may be present in the data^23–26^. Significant progress in magnetic data processing and interpretation techniques has significantly refined structural analysis and the identification of porphyry magnetic signatures, aiding in the precise identification of high mineral potential sites^27^. Thus, we employed aeromagnetic techniques as a tool for mineral exploration to determine magnetization contrasts between rock types, geological contacts, dykes, and structural features.

Although several previous studies have addressed iron mineralization in the El-Bahariya depression using either multispectral remote sensing or conventional aeromagnetic interpretation in isolation, the present study introduces a fully integrated, multi-sensor exploration framework that has not previously been applied to this district. Our aim will shed the light on the following: (i) integration of hyperspectral PRISMA data with high-resolution aeromagnetic analysis, for the first time, for the Bahariya iron ore deposits; (ii) automated lithological mapping utilizing machine learning algorithms (Random Forest and Support Vector Machine) applied to a stacked ASTER + Sentinel-2 dataset; (iii) employs advanced edge-detection filters (THGED and MGTHG) together with CET-GA and CET-PA techniques to refine the structural framework controlling iron mineralization; and (iv) integrates whole-rock geochemistry to discriminate economic low-Mn ironstones from non-economic Mn-rich carbonates and barren siliceous rocks, thereby linking the spectral and magnetic signatures directly to ore-grade material. This integrated framework allowed the detection of previously unrecognized high-potential mineralization zones beyond the boundaries of the existing mines, providing a replicable exploration pattern for similar sedimentary-hosted iron deposits in arid regions worldwide.

## Geology of the study area

El-Bahariya is a large depression in the Western Desert of Egypt located ca. 270 km southwest of Cairo and ca. 180 km west of the Nile Valley (Fig. 1). The study area is delineated by latitude 28°17’57"N to 28°31’06"N and longitude 28°56’14"E to 29°15’35"E, covering an area of ca. 800 km² (Fig. 1c). Economic iron ore deposits with an average iron content of 47.6 wt% Fe have been documented in El-Bahariya^28–30^.

The ore is confined to the northern part of the depression, covering approximately 11.7 km² with an average thickness of 9 m^31^. Iron ore mineralization is restricted to the Lower Middle Eocene carbonate sequences of the Naqb and Qazzun formations^32^, distributed in the northeastern plateau across four principal mine areas: (1) Nasser and (2) Ghorabi (combined 3.5 km²); (3) El-Gadidah (15 km²); and (4) El-Harrah (2.9 km²). The El-Bahariya Depression is deformed by a NE-trending right-lateral wrench fault system (Fig. 1b) associated with doubly plunging anticlines and E–W to NW–SE extensional faults^33–35^.

**Fig. 2:**
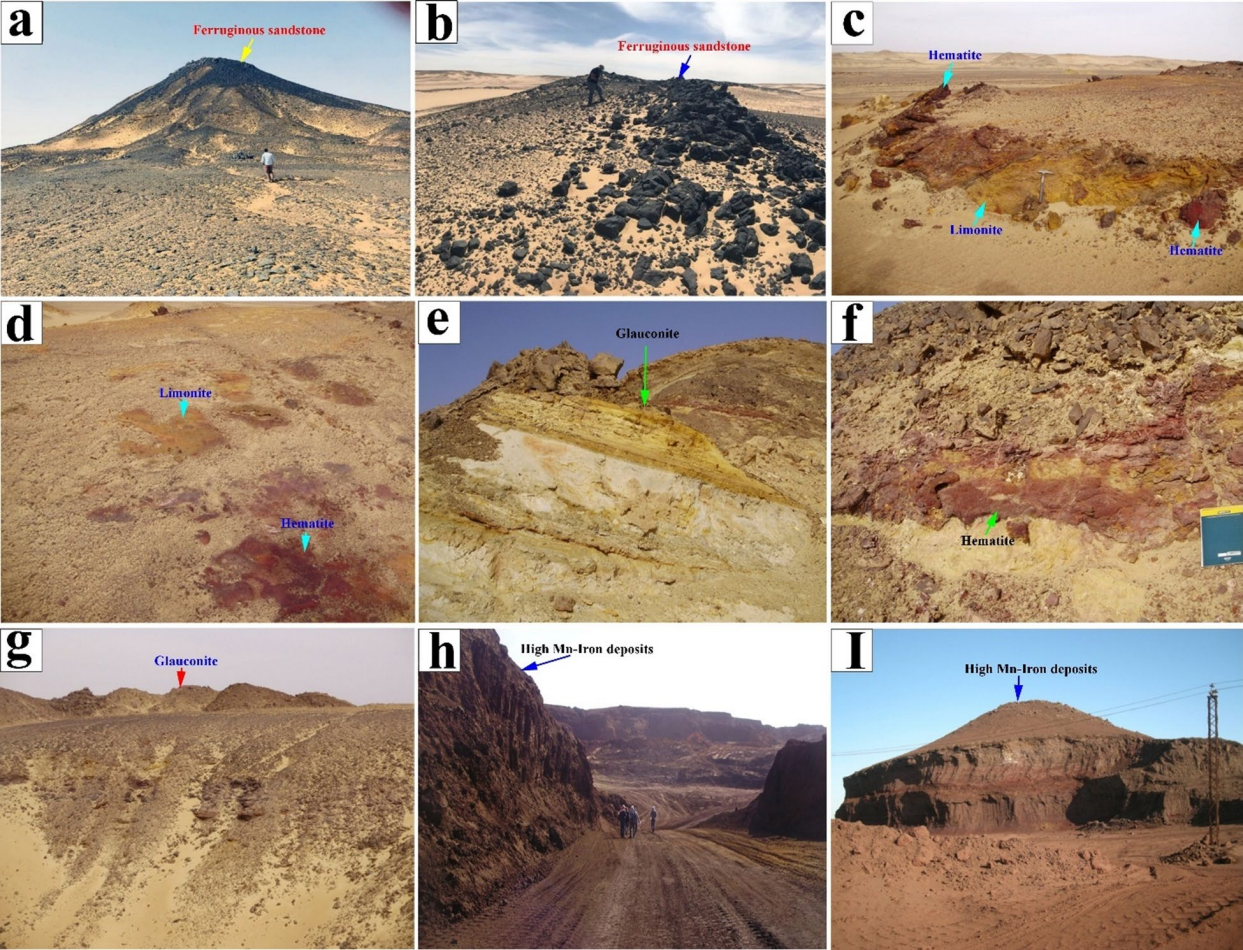
Field photographs documenting the lithological and mineralogical characteristics of the El-Bahariya iron ore deposits and their host formations. (**a**) Ferruginous sandstone and ferricrete duricrust capping a residual conical hill, showing uneconomic lateritic iron-bearing rocks of the El-Bahariya Formation. (**b**) Low-relief ridge of ferruginous sandstone, characteristic of the Cenomanian El-Bahariya Formation. **(c)** Low ridge of hematite and limonite mineralization south of Gabal Nasser mine area, with distinctive ochre-to-brown coloration reflecting Fe-oxide concentration gradients and spectral signatures diagnostic of ferric iron (hematite, Fe₂O₃) and ferric oxyhydroxide (limonite, FeO·OH) deposits. (**d**) Close-up of surface alteration and iron oxide precipitation in the Nasser area valley floor, showing brown hematite and yellow limonite patina with color-graded Fe enrichment. (**e**) Fractured and folded glauconitic siltstones interbedded with iron ore lenses in the eastern Harah sector. (**f**) Stratigraphic succession in the eastern Harah locality, showing glauconitic (“green clay”) siltstones interspersed with iron-rich bedded zones (30–50 cm thick), overlain by a quartz sandstone cap, exemplifies the Naqb Qazzun Formation ore sequence. (**g**) Topographic expression of glauconitic rock outcrops in the Ghorabi area, mantled by debris and siliciclastic sedimentary cover. (**h**,** i**) Outcrop of high-manganese ironstone in the western plateau sector, exposed at the surface without sedimentary cover, displaying burgundy-hued Mn-oxide mineral assemblages and oxidized iron-bearing surfaces characteristic of supergene enrichment. These photos are our own, and we agreed to publish them.

Ferruginous sandstone, mudstone, and concretionary iron oxide bands constitute the dominant exposed Cenomanian succession of the El-Bahariya Formation (Fig. 2a, b). High-standing ferricrete duricrust caps residual hills and low-relief ridges of Cenomanian ferruginous sediments, particularly in the El-Heiz area south of the depression (Fig. 2a, b). These ferricrete deposits represent paleoerosional (lateritic) weathering surfaces composed primarily of uneconomic ferruginous sandstones, previously and incorrectly classified as ore-grade material^36,37^. Paleokarstification processes have resulted in thin encrusting ferricrete and silicrete layers overlying the exposed Lower to Middle Eocene carbonate sequences of the Naqb Formation^38^.

The El-Gadidah mine area, an oval-shaped depression spanning up to 15 km², is situated within degraded karst cone hills of the Lower Middle Eocene carbonate sequences. The central sector rises to 254 m above sea level, while the surrounding lowlands descend to 198 m. The stratigraphic succession comprises (base to top): Cenomanian El-Bahariya Formation sandstones and sandy clays, overlain by the main Lutetian iron ore successions of the Naqb-Qazzun sequences, and capped unconformably by late Lutetian-Bartonian glauconitic sediments with lateritic ironstone interbeds of the Hamra Formation (Supplementary 1–1).

The ore sequence exhibits marked thickness variation: maximum thickness (up to 35 m) is attained in the Eastern and Western Wadi sectors, while the elevated central plateau shows significantly reduced thickness (11 m). The iron ore succession comprises a basal pisolitic–oolitic ironstone unit followed by bedded karst (lateritic) iron ores intercalated with ferruginous mudstones (Fig. 2d, e; Supplementary 1–1)^4,39–42^. Field observations (Fig. 2c, f) document the distinctive ochre-to-brown coloration of hematite and limonite deposits in low ridges south of Gabal Nasser, with color-graded variations reflecting Fe-oxide concentration profiles and corresponding to spectral absorption features diagnostic of ferric iron minerals (hematite, Fe₂O₃) and ferric oxyhydroxides (limonite, FeO·OH) identified by hyperspectral analysis (see Sects. 4 − 1).

The Ghorabi–Nasser locality exposes a complementary stratigraphic succession (Fig. 2d, e; Supplementary 1–2) beginning at the base with the El-Bahariya Formation (grey to yellowish grey clayey sediments overlain by glauconitic sandstone beds, 20 cm to 1 m thick, interspersed with iron-rich bands and concretions; total glauconite-bearing unit thickness ca. 4 m) and transitioning upward to the Naqb-Qazzun sequence. Field photographs (Fig. 2d, e) illustrate the glauconitic (“green clay”) siltstones characteristic of this succession, intercalated with iron ore lenses (30–50 cm thick) and overlain by quartz sandstone caps, confirming the lithofacies boundaries delineated by remote sensing and machine learning classification (see Sect. 4.1). Fractured and folded geometries of glauconitic units observed in the field (Fig. 2e) provide evidence of ductile deformation associated with late-stage compressional structures (NE–SW and NW–SE trending faults) identified in aeromagnetic lineament analysis, substantiating the structural control on ore distribution proposed by earlier workers and confirmed by this study.

The Hamra Formation overlies ore successions unconformably with late Lutetian-Bartonian glauconitic sediments interbedded with lateritic ironstone. The Lower and Upper Hamra Formation units, composed of glauconitic siltstones and mudstones, are prominently exposed as conical hills and ridge systems in the Ghorabi area (Fig. 2g), often mantled by siliciclastic debris and younger sedimentary cover. The topographic expression and surface manifestation of these glauconitic units are successfully discriminated by spectral unmixing techniques (Minimum Noise Fraction analysis and semantic segmentation) and machine learning classification (see Sects. 4 − 1), demonstrating the importance of multi-scale remote sensing and algorithmic approaches in mapping covered or partially exposed lithologies. Figure 2h, i, shows the high-manganese iron ore deposits outcrops in the area.

## Materials and methods

### Remote sensing data

In the current study, both multispectral (Sentinel-2 and ASTER) and hyperspectral (PRISMA) satellite datasets have been utilized to map various types of iron oxides in the El-Bahariya area. The multispectral Sentinel-2 has a higher radiometric resolution (12-bit) compared to ASTER data (8-bit), while ASTER data has a slightly higher spectral resolution (14 spectral channels) than Sentinel-2. The narrower bandwidth of the ASTER SWIR region provides more accurate spectral identification of rocks and minerals compared to other multispectral sensors^5–7,9–11,43–50^. ASTER measures both reflected and emitted radiation in the spectral range from 0.52 to 2.43 μm with three different spatial resolutions [e.g., 7, 9–11]. We used four hyperspectral L2D PRISMA datasets, which are cloud-free and were acquired on 15 July 2021 and 1 August 2020 and downloaded from the mission website (www.prisma.asi.it*).* As described in the specifications for PRISMA (see www.prisma.asi.it*)*, L2D PRISMA products are atmospherically corrected images based on the ASI standard data processing chain. Bad bands recorded in SWIR (bands 86–104, 156–173) were removed as a preprocessing step. For more details (see Supplementary 1).

Using the Sentinel-1 radar dataset, the structural features associated with alteration and iron-rich oxides in the El-Bahriya area were detected. The Sentinel-1 was acquired on March 31, 2024, and accessed through the ASF Data Search Vertex (https://search.asf.alaska.edu/) with Id (S1A_IW_GRDH_1SDV_20240331T160449.

_20240331T160518_053228_067358). The dataset comprises single-polarization acquisitions (VV or HH) in Wave mode, whereas all other interferometric wide-swath modes provide dual-polarization data (VV + VH or HH + VH). We used the Enhanced Lee filter to reduce speckles and enhance the geological contacts in the Sentinel-1 image. The combined polarization (VV + VH) was generated using band math in ENVI. Principal component analysis (PCA) was subsequently applied to VV, VH, and the derived (VV + VH) dataset, producing PC1, PC2, and PC3. Surface lineaments were extracted using the LINE module of PCI Geomatica, applied to the PC1 dataset. The rose diagram was produced using the ROCKWOR software, version 2018.

### Image processing techniques

#### Spectral band indices

The spectral band index is one of the most powerful imaging techniques for hydrothermal alteration mapping [e.g., 7, 9–11]. The presence of high digital number values within a given scene is indicative of spectral signatures that are analogous to those of the specific materials for which the values were designed to map^7,9–11,51^. In this study, various mineral indices were applied to multispectral (Sentinel-2 and ASTER) and hyperspectral PRISMA datasets to detect iron-rich oxides and ferrous minerals in the El-Bahriya area.

#### Principal component analysis (PCA)

PCA is a multivariate statistical technique used to reduce data redundancy by transforming the original data into new principal component axes, producing an uncorrelated image with much higher contrast than the original bands^52^. The first three higher-order principal components (PC1, PC2, and PC3) of the input spectral bands often account for over 97% of the spectral information, while lower-order components contain low signal-to-noise ratios^5^. Higher-order principal components provide subtle information about minerals and rock types that are spatially dominant in the image, while lower-order components can capture some spectral information that is not dominant in the image. In this study, the PCA transformation has been applied to 9 bands of Landsat-8 (VNIR, SWIR, and TIR). The PCs loadings were calculated based on correlation rather than the covariance matrix.

#### Minimum noise fraction (MNF)

The (MNF) transform is used to determine the inherent dimensionality of image data. It segregates the noise from the data and reduces the computational requirements for the subsequent processing^53^. This technique is used as a pre-processing tool in mineral mapping [e.g., 51]. In this study, the MNF technique has been applied to ASTER, as well as to stacked ASTER and Sentinel-2 datasets, to extract the image endmembers.

#### Pixel purity index (PPI) algorithm

PPI is a method of obtaining the most “spectrally pure,” or extreme, pixels in multispectral or hyperspectral scenes and can be run on the previously extracted output of MNF images. It is estimated by repeatedly projecting n-dimensional (n-D) scatter plots onto a random unit vector. The PPI image is constructed where each pixel value matches the number of opportunities that a pixel was registered as extreme. The outputs of the PPI are used as input into n-D Visualize [e.g., 5]. The extracted end-members are characterized using a spectral analysis procedure. These end-members (spectra) for ASTER are also compared to the spectra of the field samples collected using the Portable Mineral Identifier Spectroradiometer (ASD) and their existing reference from the USGS spectral library. The resampled USGS mineral library is utilized through spectral analysis to identify the material of the end-members extracted from the pure pixel index images using the Spectral Angle Mapper (SAM) classification algorithm.

#### ASD Hyperspectral data processing

Reflectance spectra are measured for the collected iron samples of El-Bahariya (hematite and limonite) in the spectral range of 350–2500 nm using an ASD Fieldspec 3 spectroradiometer. ASD has a spectral resolution of 3 nm in the spectral range of 300–1000 nm and 10 nm in the spectral range of 1000–2500 nm [e.g., 6, 7, 9–11]. These spectra are analyzed with reference to the spectral library profiles to detect the spectral diagnostic absorption features of iron samples. Reflectance spectra are derived by normalizing the spectral radiance of the samples with respect to a white reference^54–56^.

### Fieldwork and geochemical analysis

Fieldwork was conducted in two stages. The first stage involved collecting representative iron ore samples from the main old mine areas, which were valuable during the remote sensing data processing stages, as laboratory spectra were collected from these samples for the mineral mapping processes. The second stage of fieldwork has been completed, involving the collection of verification samples from some of the selected sites, which were extracted from the remote sensing data analysis. These sites have been selected based on their potential iron ore concentrations as identified from remote sensing data analysis. Several samples were collected during the second stage of the fieldwork, including nine representative samples (iron stones, carbonatized rocks, and silicic rock), which were geochemically analyzed for both major and trace elements. All fieldwork activities complied with institutional guidelines and applicable national regulations governing geological field investigations. No permission was needed to conduct fieldwork.

The chemical analyses of nine rock samples were conducted at the Analysis and Consulting Unit, National Research Center, Cairo, Egypt, using a PANalytical Axios Advanced Sequential WD-XRF Spectrometer, Model 2005. The Elemental Analysis by Wavelength Dispersive X-Ray Fluorescence Spectrometry test method was applied.

### High-resolution aeromagnetic data

The aeromagnetic data collection produced a total magnetic intensity (TMI) map, with a geographic inclination of 41.5° North and a declination of 1.86° East, recorded by Aero Service in 1984. This raw TMI map was digitized and converted into an X, Y, and Z file. The data was then gridded using the Geosoft software, resulting in the creation of the TMI grid. The completed TMI map is displayed at a scale of 1:50,000. A critical step in data processing was applied to the gridded total magnetic intensity map using the Fourier transform process described by^56^. This procedure generated a map aligned to the North Magnetic Pole, positioning the magnetic anomalies directly above their sources. The resulting reduced-to-pole (RTP) data proved invaluable for conducting a precise structural complexity analysis of the studied region.

After applying the RTP correction, the upward continuation was employed to attenuate short-wavelength anomalies^57^. This technique offers multiple benefits, such as minimizing high-frequency noise and accentuating regional magnetic anomalies. The produced maps display the distribution sources of magnetic susceptibility, emphasizing those at various depths that are notably important. The representation goal is to map the varying depths of magnetic sources, with each map concentrating on distinct depth ranges to offer a thorough insight into subsurface magnetic anomalies. As part of this study, the upward continuation technique was applied to the magnetic data at depths of 1 and 3 km. This approach revealed magnetic sources at depths of 0.5 and 1.5 km, respectively, thereby improving the detection of magnetic anomalies and supporting subsurface structural investigations^27^.

#### High-precision edge detection

Edge detection is a widely used technique in geophysical exploration for identifying and mapping features such as faults, boundaries, dykes, and mineral deposits^19,58,59^. These techniques play a crucial role in interpreting potential field data, with the primary goal of accurately defining the boundaries of magnetic sources and structures^60–62^. Two enhanced techniques have been applied to analyze magnetic data, as follows:

(i) *Enhanced Tilt Angle of the Horizontal Gradient (THGED)*.

The THGED filter is a new normalized detector that offers a more reliable edge detection compared to conventional methods, as outlined by^63^:

1 $$\:THGED=atan\:\frac{{HGA}_{Z}-\:\sqrt{{{HGA}_{X}}^{2}+{{HGA}_{Y}}^{2}}}{\sqrt{{{HGA}_{X}}^{2}+{HG{A}_{Y}}^{2}+{HG{A}_{Z}}^{2}}}$$

Where

2 $$HGA=\:\sqrt{{{F}_{X}}^{2}+{{F}_{Y}}^{2}}$$

The main purpose of the THGED filter is to identify abrupt changes in magnetization. The peaks in the THGED filter outline the boundaries of the source bodies. One of the main strengths of the THGED lies in its ability to define the edges with exceptional accuracy and sharpness.

(ii) *Modified Gudermannian function of total horizontal gradient (MGTHG)*.

The advanced MGTHG filter, introduced by^64^, utilizes the Modified GF^65^ to outline the horizontal boundaries of potential field sources at different depths. The MGTHG improves the precision of boundary detection by sharpening the resolution of potential field data. The filter is represented as:

3 $$\:MGTHG=\:\frac{2}{\pi\:}\:atan\:\left\{sinh\left(\frac{{HGA}_{Z}+{HGA}_{Z}-\sqrt{{{HGA}_{X}}^{2}+{{HGA}_{Y}}^{2}}}{\sqrt{{{HGA}_{X}}^{2}+{{HGA}_{Y}}^{2}}}\right)\:\right\}$$

The MGTHG filter’s peaks clearly define the edges of the casutive bodies. Its amplitude varies from − 1 to + 1 radians. One critical advantage of the MGTHG filter is its ability to sharply and accurately outline these boundaries. Unlike many modern high-resolution filters, the MGTHG filter maintains consistent resolution in its results, unaffected by any parameters chosen by the user^61,66–68^.

### Euler depth estimation method (EUD)

Thompson^69^ first proposed the EUD as an automated method to identify the location and depth of magnetic sources within realistic magnetic datasets and profiles. Reid^70^ later modified this technique for use with magnetic-grid data. Euler deconvolution utilizes potential field derivatives to estimate the depths of subsurface magnetic sources^71^. This technique effectively identifies subsurface boundaries and reveals fault trends, with its relationship of homogeneity mathematically expressed by the following equation:

4 $${\rm \partial T/\partial X (X - X_0) + \partial T/\partial y\:(y- y_0) +\partial T +\partial z/\partial z(z- z_0) = SI (B-T)}$$

The structural index (SI) expresses the structural features, while (B) denotes the base level of the field, and (T) refers to the observed magnetic field at specific x, y, and z coordinates. This method was applied to the RTP magnetic dataset to identify structural patterns associated with altered zones and assess their depth.

#### Apparent susceptibility mapping (AMS)

Mapping magnetic susceptibility involves an analytical approach where the measured magnetic data is transformed directly into a spatial distribution of susceptibility contrasts^72^. Apparent susceptibility mapping (AMS) employs a series of linear filtering, including reduction to the pole and downward continuation, to approximate the depth of the magnetic source. This process adjusts for the geometric influence of a vertically oriented, square-ended prism. The outcome is then normalized by the total magnetic intensity to derive the susceptibility distribution^73^. The derivation of the apparent susceptibility map (AMS) rests on three key premises: that the data have been adjusted to correct the IGRF, that magnetization occurs exclusively by induction, and that every magnetic signature can be modeled as an assembly of vertically oriented, square-based prisms extending infinitely in depth. To estimate the apparent susceptibility contrast, the magnetic data were downward continued to a depth of 100 m. The reduction to the pole was performed using geomagnetic parameters recorded in 1984, which included a field strength of 42,000 nT, an inclination angle of 41.55°, and a declination of 1.88° relevant to the study area.

### Center of exploration and targeting grid analysis technique (CET-GA)

The CET-GA grid analysis technique enhances the texture of magnetic images, making it easier to identify areas with structural complexity that are valuable for exploration goals. The structural complexity (SC) method helps pinpoint zones with a high prospect of hosting mineral deposits^74^. The CET-GA technique analyzes image textures to detect structural features, such as contacts, boundaries, and edges, using a step-by-step approach. It begins by applying the standard deviation to assess magnetic variations. Then, it utilizes phase symmetry analysis to identify ridges, and amplitude thresholding is used to extract the ridge line segments. Next, skeletonization (line thinning) is applied to generate axial lines. The final step involves conducting a complexity analysis to produce a density map that emphasizes regions with frequent contact occurrences^75–77^.

#### Center of exploration and targeting porphyry analysis technique (CET-PA)

**Fig. 3:**
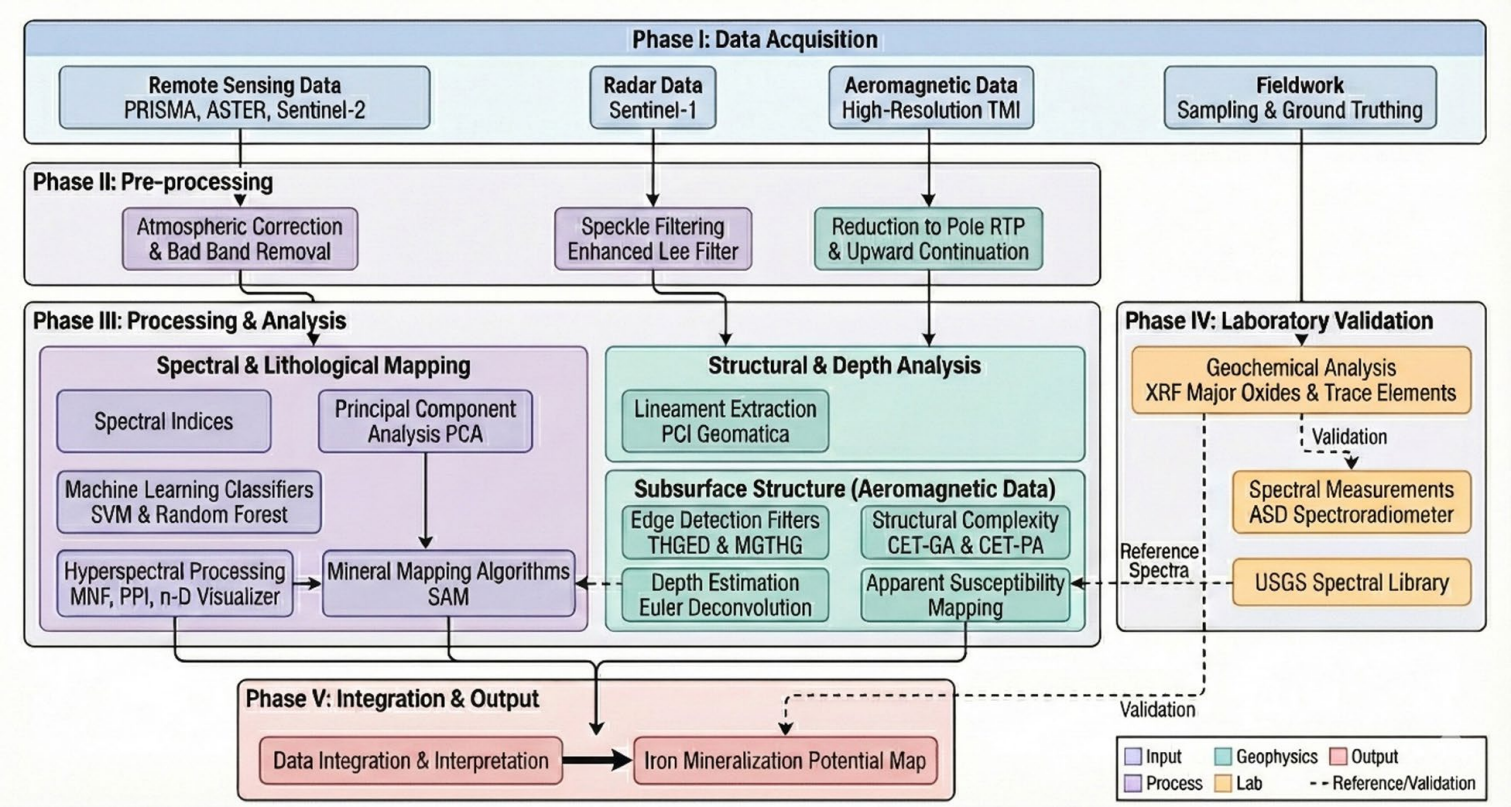
Flowchart of the proposed methodology used in this study.

The CET Porphyry method utilizes the Circular Feature Transform (CFT) as an initial step to detect and map circular and semi-circular anomalies^78^. Following this, the technique identifies the central points of circular highs and lows. The Amplitude Contrast Transform (ACT) is then applied, allowing each circular feature to be represented by a distinct “halo,” which emphasizes its outer edge. The final results include a database file that logs the locations of the detected circular features, along with their radial symmetry strength and optimal radius (provided in both cells and meters). Additionally, a polygon file is created, which defines the boundary corresponding to the highest radial symmetry around each detected center, facilitating a clear view of the spatial extent of these circular features^74,79^. The methodology adopted in the current study is presented in Fig. 3.

## Results

### Automated lithological mapping using machine learning algorithms

**Fig. 4:**
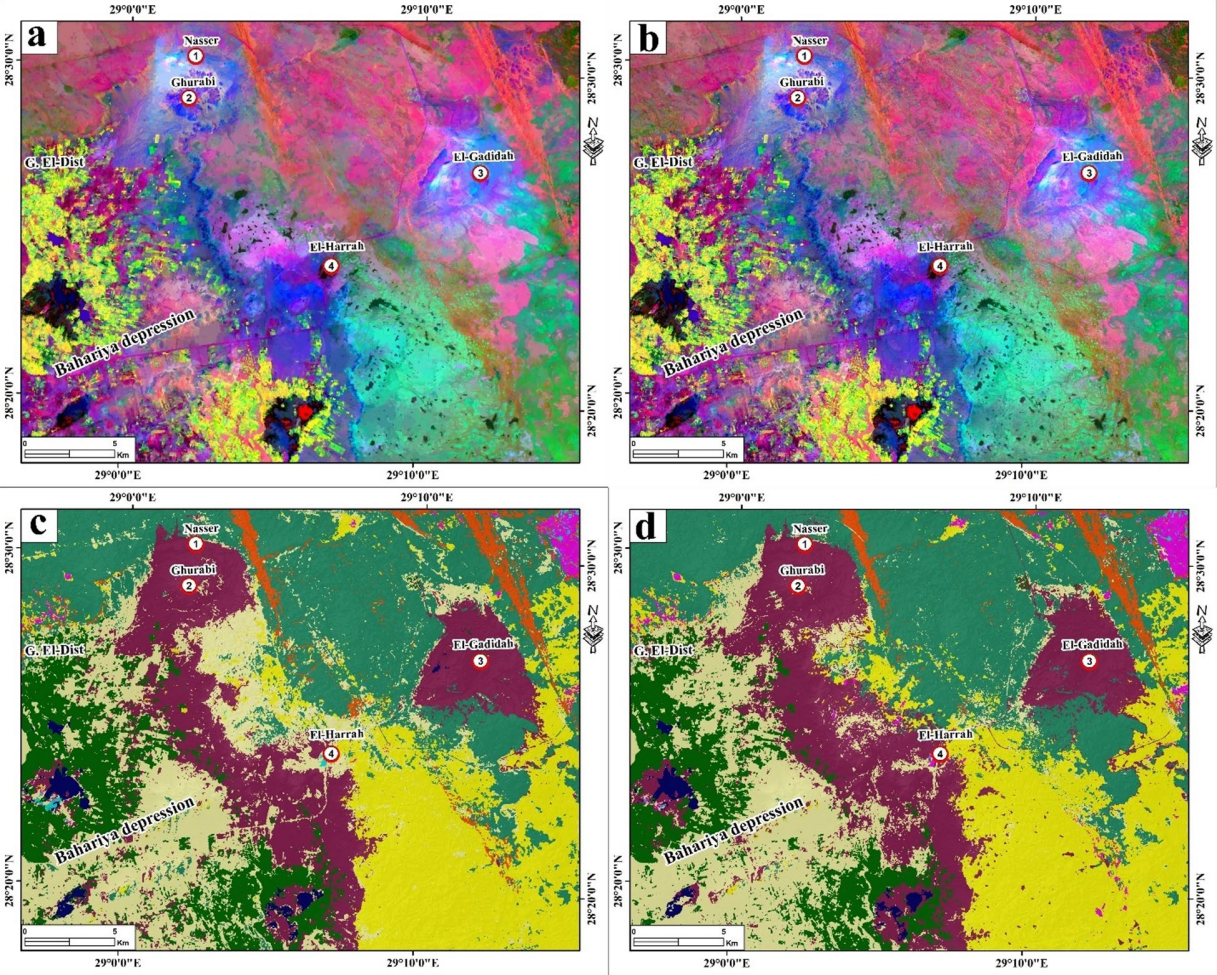
**(a)** Minimum Noise Fraction (MNF) composite (b4, b3, b2 as RGB) of stacked ASTER + Sentinel-2, highlighting principal spectral variability and dimensionality reduction for endmember extraction. (**b**) Semantic segmentation output showing lithological class boundaries delineated through unsupervised/semi-supervised learning, providing visual interpretation of spectral class distributions before supervised classification. Automatic lithological detection using machine learning classification algorithms applied to ASTER + Sentinel-2 data using RBF support vector machine (**c**) and Random Forest applied (**d**). Created by QGIS Desktop 3.36.3 software; https://qgis.org/project/visual-changelogs/visualchangelog336/ and ArcGIS Desktop 10.8. https://www.esri.com/en-us/arcgis/ 289 products/arcgis-desktop/overview.

Training sets comprising 356,555 pixels distributed across nine lithological classes (Bahariya Formation, Qazzun Formation, Naqb Formation, Lower Hamra Formation, Upper Hamra Formation, Rodwan Formation, Iron Ore Deposits, Agriculture lands, Water bodies) were constructed from previous geological maps [e.g., 35], Minimum Noise Fraction (MNF) composite imagery (b4, b3, b2 as RGB) derived from stacked ASTER and Sentinel-2 data (Fig. 4a), and semantic segmentation output (Fig. 4b). The full pixel set was divided into 80% training (285244 pixels) and 20% validation (71311 pixels) subsets for unbiased performance assessment. Two supervised machine learning algorithms, Support Vector Machine (SVM, RBF kernel) and Random Forest (RF), were applied to the stacked ASTER and Sentinel-2 multispectral dataset to classify lithological units across the entire El-Bahariya depression.

#### Support Vector Machines

The RBF-kernel Support Vector Machine, a supervised algorithm that optimizes a nonlinear decision hyperplane by maximizing inter-class margins using critical support vectors^80^, was trained on the 80% training pixel set and evaluated on the 20% validation set. This study employed an RBF-SVM kernel, which is effective for automated lithological mapping^6,10–12,81,82^. SVM achieved an overall accuracy of 90.37%, Kappa accuracy of 88.31%, and a mean F1-score of 93.71% (Table 1). Class-specific performance (F1-scores) revealed excellent discrimination across most lithofacies: Naqb Formation (96.74%), Lower Hamra Formation (99.25%), Upper Hamra Formation (97.12%), Water Body (99.98%), and Rodwan Formation (95.51%), with the strongest detection of carbonate-hosted and glauconitic units. Iron Ore Deposits achieved F1 = 89.30%, with precision of 93.09% and recall of 85.79%, indicating that SVM successfully identified 85.8% of true ore pixels while maintaining a false-positive rate of ~ 7% (Table 1). Moderate performance was observed for the Bahariya Formation (85.05% F1) and Qazzun Formation (85.60% F1), attributed to spectral overlap between ferruginous sandstones (BF) and carbonate-hosted ore (QF), evident in confusion matrix entries showing 6150 BF pixels misclassified as QF and 13,394 QF pixels misclassified as BF. Agriculture lands achieved 97.65% F1, indicating excellent non-geological class separation. Principal sources of misclassification were: (1) BF confused with QF and IOD due to shared iron-oxide absorption features; (2) QF confused with BF due to lithofacies gradation; and (3) minor Rodwan (basement) confusion with NF and UHF at geological contacts.

#### Random Forest

Random Forest, a decision-tree ensemble learning algorithm^83^, was trained and validated on the same pixel partitioning as SVM. RF achieved an overall accuracy of 91.73%, a Kappa accuracy of 89.99%, and a mean F1-score of 93.98%, representing improvements of 1.36%, 1.68%, and 0.27% respectively, over SVM (Table 1).

**Table 1:** Confusion matrices, overall, and class-based statistics for SVM and RFs based on ASTER+Sentinel-2 data. Abbreviations: Baharyia Fm (BF), Qazun Fm (QF), Naqb Fm (NF), Lower Hamama Fm (LHF), Upper Hamama Fm (UHF), Rodwan Fm (RF), Iron ore deposits (IOD), Agriculture lands (Ag), Water body (Wb).

SVM	BF	QF	NF	LHF	UHF	RF	IOD	Ag	Wb	Sum	Precision%	Recall%	F1-score%
BF	81,991	6150	30	0	0	0	5490	0	0	93,661	87.54	82.7	85.05
QF	13,394	60,955	9	27	0	0	491	0	0	74,876	81.41	90.25	85.6
NF	520	70	61,574	0	0	2003	536	0	0	64,703	95.16	98.38	96.74
LHF	0	219	0	18,449	2	0	0	0	0	18,670	98.82	99.68	99.25
UHF	0	0	0	15	4587	13	0	0	0	4615	99.39	94.95	97.12
RF	0	8	598	17	242	18,395	0	0	0	19,260	95.51	90.12	92.74
IOD	3080	140	41	0	0	0	43,963	0	0	47,224	93.09	85.79	89.3
Ag	157	0	338	0	0	0	762	26,081	0	27,338	95.40	100	97.65
Wb	2	0	0	0	0	0	0	0	6206	6208	99.97	100	99.98
Sum	99,144	67,542	62,590	18,508	4831	20,411	51,242	26,081	6206	356,555	**OA = 90.37**		
Overall Accuracy%											**90.37**		
Kappa Accuracy%											**88.31**		
Mean F1 Accuracy%											**93.71**		
RF	**BF**	**QF**	**NF**	**LHF**	**UHF**	**RF**	**IOD**	**Ag**	**Wb**	**Sum**	**Precision%**	**Recall%**	**F1-score%**
BF	86,504	4698	49	0	0	5	2405	0	0	93,661	92.36	90.93	91.64
QF	6682	61,097	0	31	0	0	7066	0	0	74,876	81.60	91.48	86.26
NF	35	31	60,186	0	0	3470	981	0	0	64,703	93.02	99.27	96.04
LHF	0	286	0	18,382	2	0	0	0	0	18,670	98.46	99.78	99.12
UHF	0	0	0	8	4566	41	0	0	0	4615	98.94	97.61	98.27
RF	0	1	353	1	110	18,795	0	0	0	19,260	97.59	84.24	90.42
IOD	1682	674	38	0	0	0	44,830	0	0	47,224	94.93	80.18	86.93
Ag	23	0	0	0	0	0	629	26,686	0	27,338	97.62	100.00	98.79
Wb	203	0	0	0	0	0	0	0	6005	6208	96.73	100.00	98.34
Sum	95,129	66,787	60,626	18,422	4678	22,311	55,911	26,686	6005	356,555	**OA = 91.73**		
Overall Accuracy%											**91.73**		
Kappa Accuracy%											**89.99**		
Mean F1 Accuracy%											**93.98**		

Class-wise F1-scores demonstrated higher discrimination across the lithological spectrum: Naqb Formation (96.04%), Lower Hamra Formation (99.12%), Upper Hamra Formation (98.27%), Agriculture (98.79%), and Rodwan Formation (97.59%), with Bahariya Formation particularly improved to 91.64% (6.59% point gain over SVM). Iron Ore Deposits achieved an F1 score of 86.93%, with a precision of 94.93% and a recall of 80.18% (Table 1). This marginally lower recall compared to SVM (a 2.4% difference) is offset by a higher precision (1.84% improvement), yielding fewer false positives at the cost of approximately 5.6% more missed ore pixels. Geologically, this trade-off is meaningful: the lower recall reflects the spectral overlap between iron-rich Bahariya/Qazzun horizons and the actual ore zones rather than a systematic bias of the algorithm, and the higher precision is preferable for exploration targeting because it minimizes the number of false-positive ore pixels reported to drill planners. Qazzun Formation F1 improved slightly to 86.26%, though significant spectral confusion with Bahariya Formation (4698 pixels) and Iron Ore Deposits (7066 pixels) continues, reflecting the gradational nature of carbonate-hosted ore horizons. The water body classification achieved an F1 score of 98.34%, with Agriculture at 98.79%, demonstrating an effective separation of non-geological classes (Table 1).

### Iron deposits mapping using multisource remote sensing datasets

Several image processing algorithms were applied to both multispectral (Landsat-8, ASTER, and Sentinel-2) and hyperspectral (PRISMA) images to understand the distribution of lithologic units containing potential iron deposits based on their spectral characteristics. Spectral band indices, principal component analysis (PCA), Pixel Purity Index (PPI), and Spectral Angle Mapper (SAM) have been used in this study to emphasize the lithological units and identify the occurrences of iron minerals distributed within and around the old mine areas.

#### Mulispectral (Sentinel-2 and ASTER) datasets

**Fig. 5:**
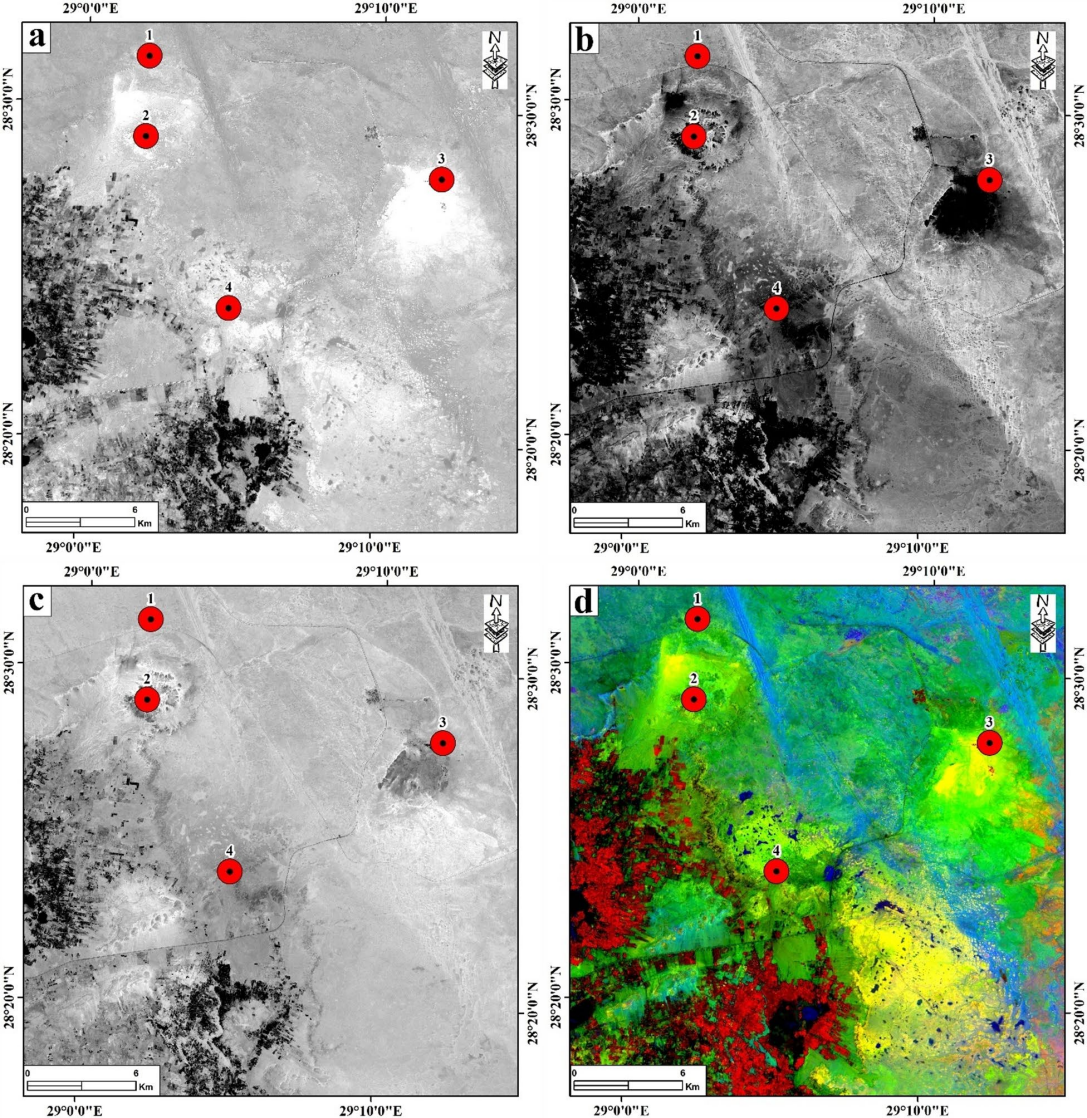
Grey scale Sentinel-2 band ratio images bands (**a**) 11/8, (**b**) 4/2, (**c**) 4/3, and (**d**) Principal component analysis (PCA) (b4, b2, b1 as RGB) of Sentinel-2. Created by ENVI v. 5.3 software; https://www.l3harrisgeospatial.com/Software-Technology/ENVI, which is mainly utilized for image processing and ArcGIS Desktop 10.8. https://www.esri.com/en-us/arcgis/products/arcgis-desktop/overview.

Analysis of Sentinel-2 band ratios (b4/b2, b3/b4, b11/b8) is chosen in order to discriminate between the iron deposits and the surrounding formations (Fig. 5). In the Sentinel-2 band ratio image bands 11/8, iron ore is represented by high-DN values (bright tones), while the background lithology appears in low-DN values (slightly darker tones) (Fig. 5a). Furthermore, the iron-rich areas are highlighted with very dark tones in Sentinel-2 band ratio image b4/b2 (Fig. 5b). The grey-scale band ratio image, using bands 3/4 (Fig. 5c), has been employed, which highlights the iron oxides ash outside the mined areas. The iron oxides are clearly visible in yellow in the Sentinel-2 PCA image (b4, b3, and b1 as RGB; Fig. 5d).

**Fig. 6:**
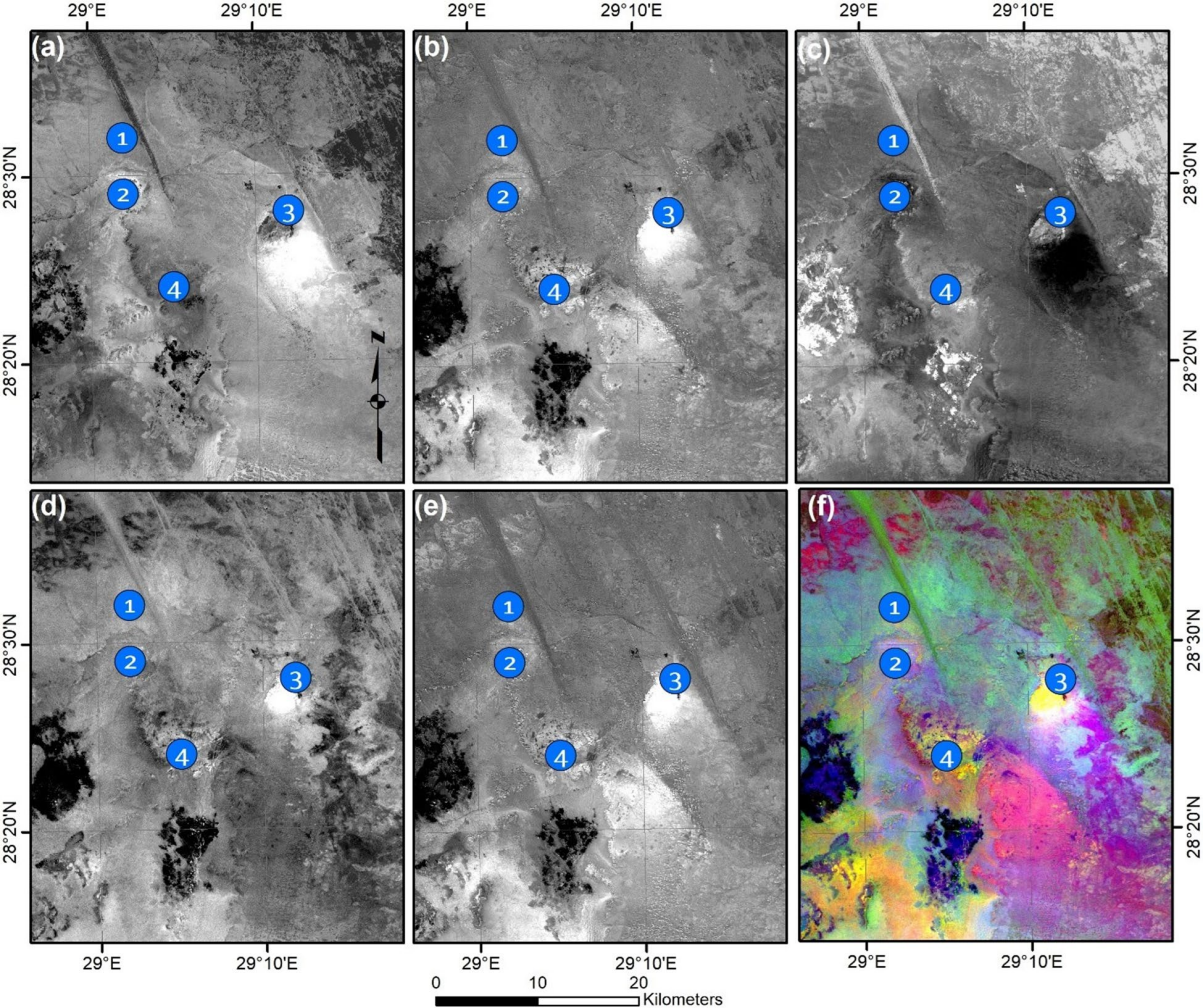
ASTER band ratio images in RGB (**a**) Ferric Iron index (bands 2/1), (**b**) Ferrous iron (bands 5/3), (**c**) Ferrous iron (bands 1/2), (**d**) Ferrous silicate index (bands 5/4), (**e**) Ferric oxides index (b4/b3), (**f**) ASTER band ratio (4/3, 5/4, 2/1). Created by ENVI v. 5.3 software; https://www.l3harrisgeospatial.com/Software-Technology/ENVI, which is mainly utilized for image processing and ArcGIS Desktop 10.8. https://www.esri.com/en-us/arcgis/products/arcgis-desktop/overview.

On the other hand, the narrower band width of the ASTER spectral bands is more accurate in the spectral identification of minerals than the Sentinel-2 dataset. Strong absorption features of the iron oxides and the surrounding rock units using ASTER (VNIR + SWIR) bands are observed in the ASTER bands 3, 6, and 8, respectively, particularly at band 8 (Supplementary 1–4). The ASTER data processing results (Fig. 6) are presented as panchromatic images, which depict variations in iron content. ASTER band ratio image bands 2/1 has been applied for Ferric iron index (Fig. 6a), which is emphasized with very dark tones, while two different ASTER band ratios (bands 5/3 and 1/2) have been applied to detect the ferrous iron with very bright tones (Fig. 6b, c). The ASTER index (band ratio 5/4) is used to discriminate between ferrous silicate (Fig. 6d). In the study area, iron silicate and glauconitic sediments constitute the upper to middle Eocene succession in the El-Gadidah and El-Harrah mines area, as part of the exposed Cenomanian clastic sequence. Ferric oxides are emphasized with a bright tone using the ASTER index (bands 4/3) image^84,85^ (Fig. 6e). The false color composite band ratio image (bands 4/3, 5/4, 2/1 in RGB) detects high iron signature areas with yellow color (Fig. 6f).

#### Hyperspectral PRISMA dataset

**Fig. 7:**
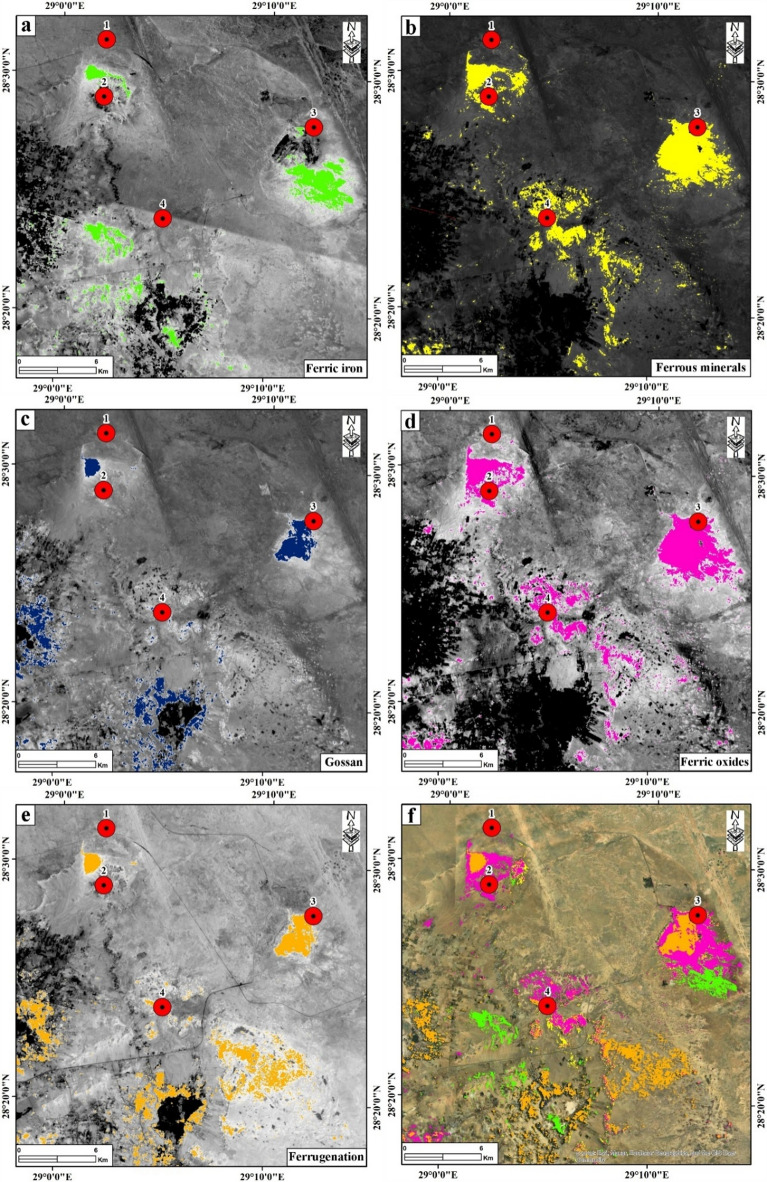
Hyperspectral PRISMA-derived mineral indices for discrimination of iron oxide, ferrous minerals, and hydrothermal alteration zones in the El-Bahariya depression. (**a**) The ferric iron mineral index (b33/b21) highlights iron-rich zones with a green color. (**b**) Ferrous iron index (b189/b47) highlighting the ferrous iron-rich zones with yellow color. (**c**) The gossan alteration index (b132/b33), highlighted in blue, emphasizes the different alteration zones. (**d**) The ferric oxide alteration index (b132/b47) emphasizes different alteration zones rich in iron, characterized by a magenta color. (**e**) Ferrugination alteration index ((b132/b33) + (b33/b21)), detecting the different alteration zones with a yellow color. (**f**) PRISMA mineral indices, showing the five types of mineral indices detected in the Baharyia area.Created by ENVI v. 5.3 software; https://www.l3harrisgeospatial.com/Software-Technology/ENVI, which is mainly utilized for image processing and ArcGIS Desktop 10.8. https://www.esri.com/en-us/arcgis/products/arcgis-desktop/overview.

**Fig. 8:**
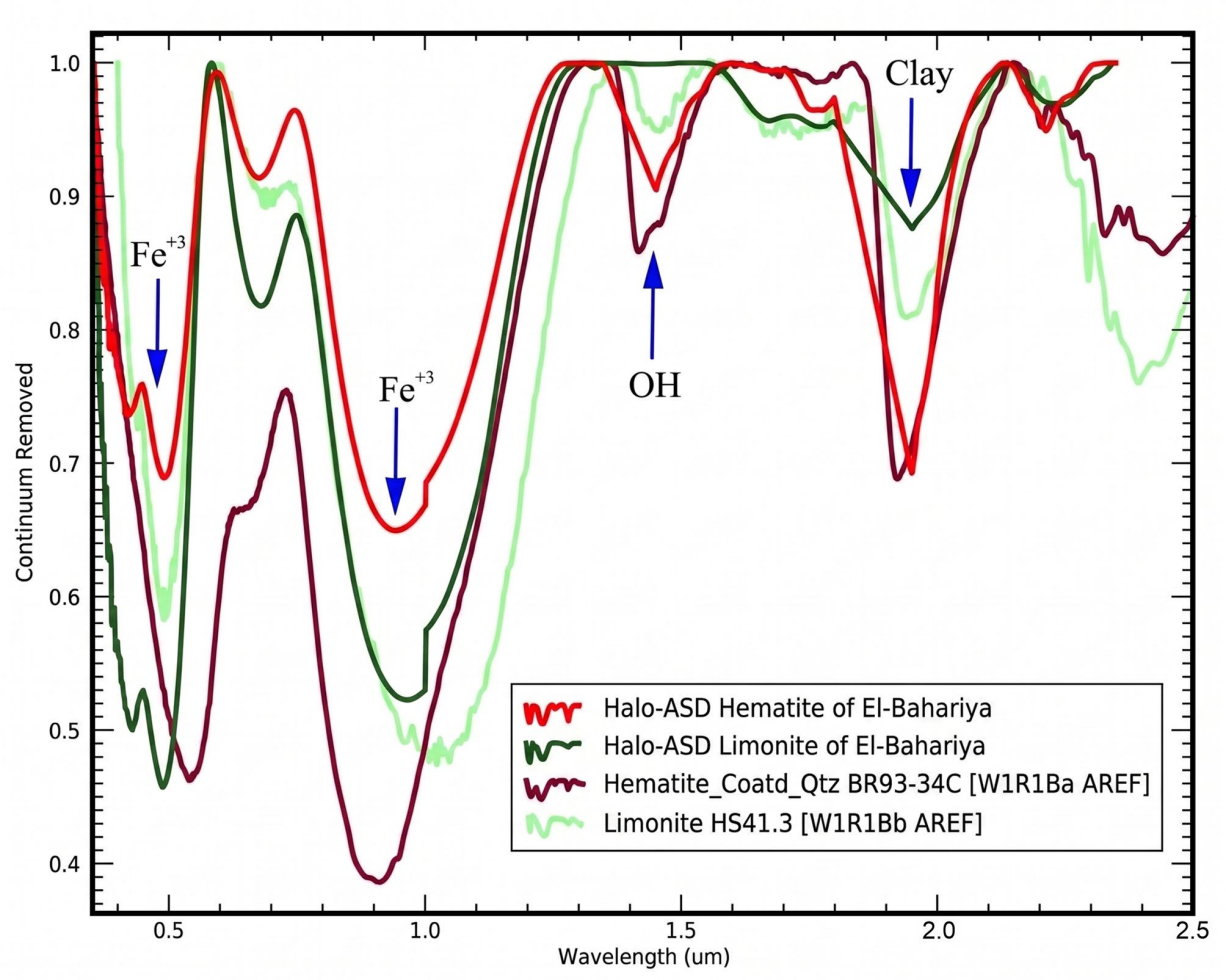
The average reflectance spectrum of the measured hematite and limonite field samples using the portable spectroradiometer (ASD). Created by ENVI v. 5.3 software; https://www.l3harrisgeospatial.com/Software-Technology/ENVI, which is mainly utilized for image processing.

Hyperspectral PRISMA data processing yielded five mineralogically diagnostic indices targeting iron oxide and hydrothermal alteration minerals characteristic of El-Bahariya’s ore deposits (Fig. 7). The ferric iron index (b33/b21), using the differential reflectance between band 33 (goethite absorption at ~ 1.04 μm) and band 21 (hematite edge at ~ 0.9 μm), successfully discriminated Fe³⁺-bearing mineral assemblages (hematite, goethite, ferric oxyhydroxide) with green color concentrated in all four known mine areas (Figs. 7a and 8). The ferrous iron index (b189/b47), sensitive to reduced iron species and magnetite absorption near 1.1 μm and reflectance in the near-infrared (band 189: 1.645 μm), yielded yellow-colored anomalies (Fig. 7b) indicating secondary iron oxide phases and magnetite-rich zones potentially formed during early diagenesis or mild hydrothermal reduction.

The gossan index (b132/b33), targeting diagnostic Al-OH and Fe-OH absorption features at 2.205 μm (band 132) and iron oxyhydroxide absorption at 1.04 μm (band 33), delineated blue-colored advanced argillic alteration zones and iron-hydroxide assemblages (Figs. 7c and 8), indicating supergene-weathered cap rocks and potential hypogene ore beneath oxidized surfaces. The ferric oxide alteration index (b132/b47) combined 2.205 μm band data (Al-OH, goethite, jarosite absorption) with near-infrared reflectance (band 47, 0.935 μm) to map magenta-colored phyllosilicate and ferric oxide-rich alteration zones (Figs. 7d and 8), consistent with secondary minerals (kaolinite, alunite) formed during acid-generating supergene and hydrothermal processes. The ferrugination index ((b132/b33) + (b33/b21)) provided a composite measure of iron oxidation intensity by summing ferric iron and iron-hydroxide spectral signatures (Fig. 7e), yielding yellow-colored zones indicating maximum iron oxide concentration and integrated primary-secondary mineralization. The five identified alteration zones in the area are shown in Fig. 7f.

In this study, fifteen measurements are collected for each sample to produce the average spectral curve of hematite and limonite field samples using ASD (Fig. 8). Spectral Feature Fitting (SFF) is an absorption-feature-based methodology. In this method, the reference spectra are scaled to match the image spectra after the continuum is removed from both datasets. The continuum represents the background reflectance of a target caused by non-selective multiple scattering from the matrix and the presence of spectrally inactive minerals. The continuum is a convex hull fit over the top of a spectrum, using straight-line segments that connect local spectral maxima (e.g., Clark et al., 1990; Cloutis, 1996; Mustard and Sunshine, 1999). SFF works best with materials that have unique and detailed absorption features, such as minerals. Therefore, SFF is used to compare the fit of image spectra to reference spectra. The investigative spectral features of both hematite and limonite samples are analyzed in relation to the library spectra of their predominant mineral constituents to understand the mineralogical significance of the spectral features using ENVI software. Continuum removal has been applied to normalize the measured iron reflectance spectra, allowing for the comparison of separate absorption features from a common baseline. For both hematite and limonite samples, it is observed that the extracted spectral behaviors of iron samples with the same ratings are spectrally similar.

#### Spectral angle mapper (SAM)

**Fig. 9:**
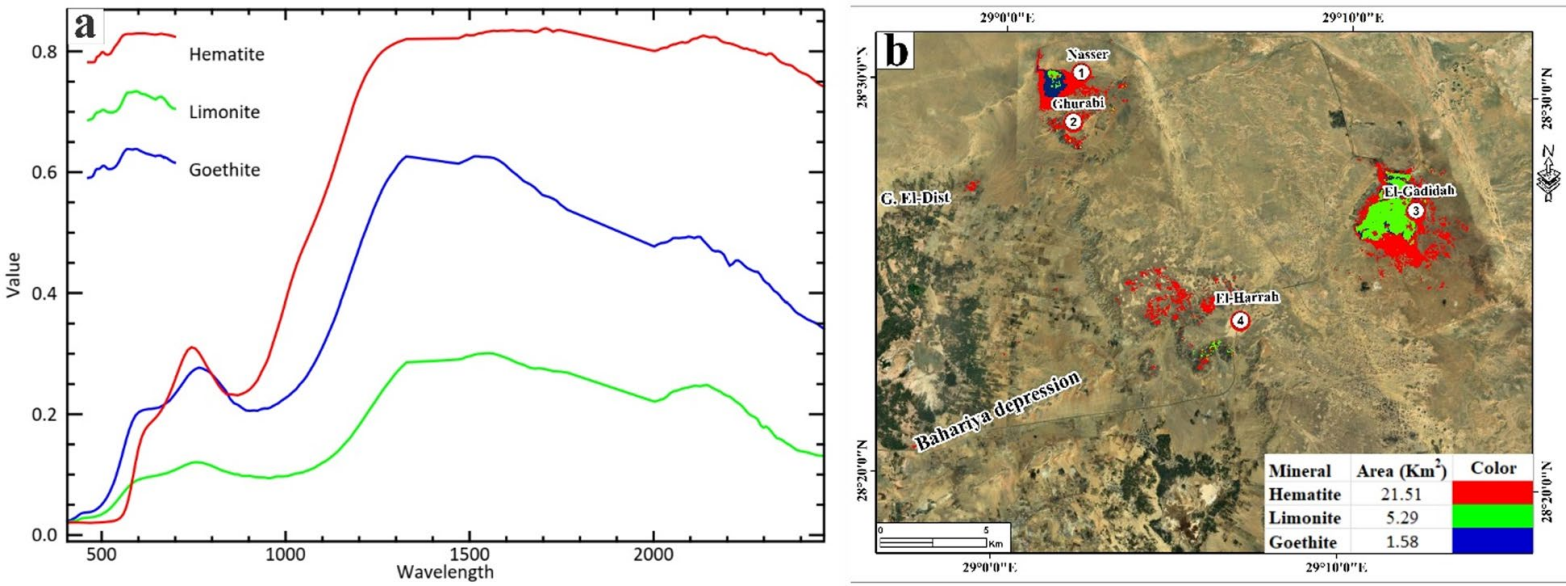
(**a**) Reference spectral curves for hematite, limonite, and goethite derived from the USGS spectral library, showing diagnostic absorption features in the VNIR and SWIR regions. (**b**) Spectral Angle Mapper (SAM) classification results applied to PRISMA hyperspectral data, delineating mineral distributions across El-Bahariya depression. Red pixels represent hematite (21.51 km²; Fe₂O₃ with absorption at 0.6 and 0.9 μm), indicating primary economic iron oxide ore. Green pixels represent limonite (5.29 km²; FeO·OH with absorption at 0.6, 0.9, and 2.2 μm), indicating supergene-oxidized ore margins and secondary enrichment surrounding hematite cores. Blue pixels represent goethite (1.58 km²; FeO·OH, subordinate phase concentrated in deep-weathering and hydrothermal alteration zones). Created by ENVI v. 5.3 software; https://www.l3harrisgeospatial.com/Software-Technology/ENVI, which is mainly utilized for image processing and ArcGIS Desktop 10.8. https://www.esri.com/en-us/arcgis/products/arcgis-desktop/overview.

SAM is a procedure that determines the similarity between a pixel and each of the reference spectra based on the calculation of the “spectral angle” between them (e.g., 50]. This method treats both the questioned and known spectra as vectors and calculates the spectral angle between them. A variety of spectral libraries is available for earth-surface materials. These are principally used for the identification of mineralogy (i.e., limonite and hematite). SAM has been applied to the PRISMA dataset, at threshold angle 0.1, to detect the distribution of both hematite, limonite, and goethite (Fig. 9). SAM classification of PRISMA successfully delineates three primary iron oxide and iron-bearing minerals across the El-Bahariya depression (Fig. 9). Reference spectral curves (Fig. 9a) derived from the USGS spectral library show diagnostic absorption features: hematite (Fe₂O₃) with sharp Fe-O absorption at 0.6 and 0.9 μm and high SWIR reflectance; limonite (FeO·OH-dominated) with Fe-OH absorptions at 0.6, 0.9, and 2.2 μm and lower overall reflectance; and goethite (FeO·OH) with similar absorption features but lowest reflectance, indicating the most heavily oxidized phase. SAM-derived mineral map (Fig. 9b) reveals: hematite as the dominant primary ore mineral, covering 21.51 km² (red pixels) concentrated in all four known mine areas; limonite as the secondary supergene-oxidized phase, covering 5.29 km² (green pixels) predominantly surrounding hematite cores, indicating the oxidized cap/transition zone; and goethite as a subordinate deep-weathering and hydrothermal alteration product, covering only 1.58 km² (blue pixels), scattered in peripheral zones. The spatial pattern of hematite cores with limonite halos is diagnostic of polygenetic ore formation with primary hydrogenous/hydrothermal precipitation overprinted by supergene oxidation. SAM-identified mineral anomalies extend well beyond existing mine boundaries into the southern and central depression areas, including the G. El-Dist zone and south of El-Harrah.

### Automatic lineaments extraction

**Fig. 10:**
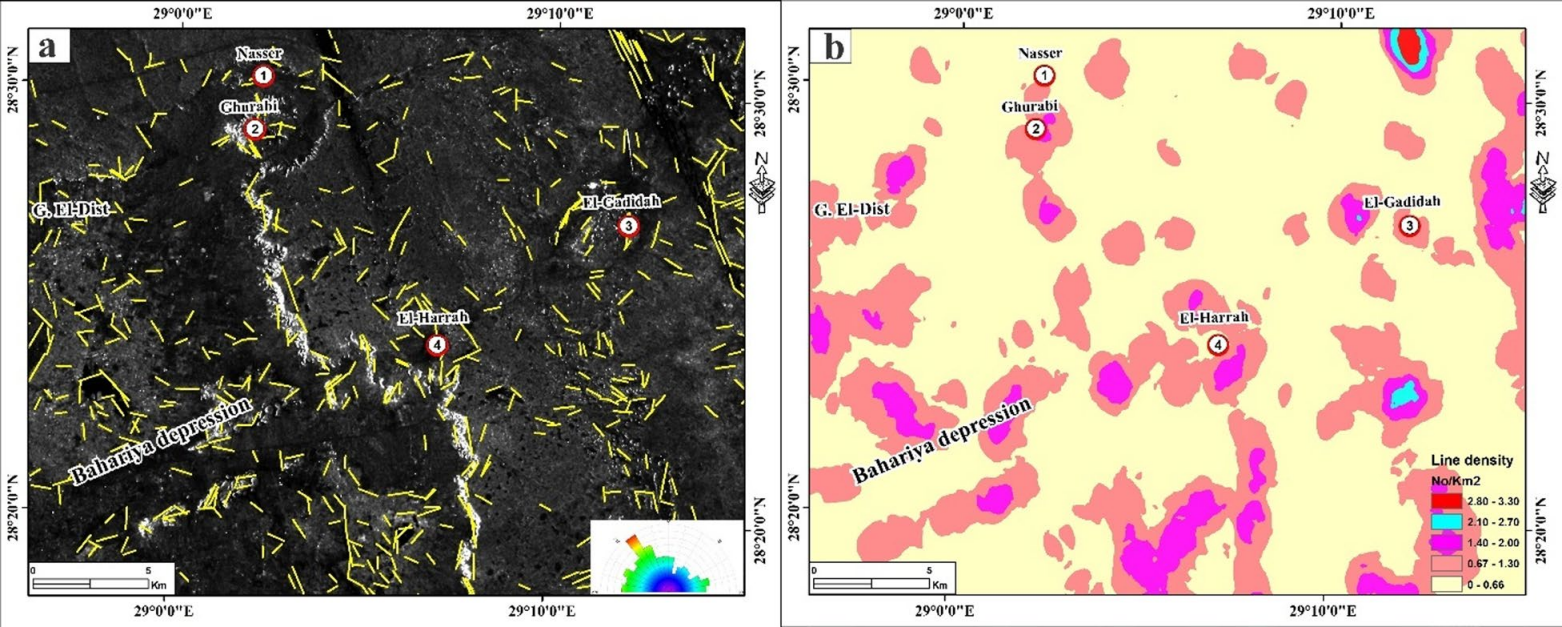
(a) Lineaments extraction and rose diagrams based on the Sentinel-1. (c, d) Lineament density map of Sentinel-1. Created by ArcGIS Desktop 10.8. software; https://www.esri.com/en-us/arcgis/products/arcgis-desktop/overview, ENVI v. 5.3. software; https://www.l3harrisgeospatial.com/Software-Technology/ENVI, Geomatica PCI software, and RCOKWORK v. 18 software; https://www.rockware.com/product/rockworks/.

Automated lineament detection provides a faster and more objective approach to geologic mapping compared to traditional manual (visual) interpretation, which is more dependent on the analyst’s experience. The rapid extraction of lineaments is essential for geologic mapping and identifying alteration zones, which are often associated with hydrothermal ore deposits located close to or within fault/fracture zones [e.g., 9–11]. Figure 10a shows the results of lineaments extracted from the Sentinel-1 dataset in the Bahariya area. The predominant surface structural lineament directions in the study area are E-W and NW-SE (Fig. 10a). These data were subsequently exported to ArcGIS version 10.8 for further analysis, including lineament density mapping using a line density module (Fig. 10b). This module depends on the frequency of the lineaments per unit area (number/km^2^). Remarkably, the lineaments and their densities are highly concentrated in specific zones within the study area, notably in the southeastern part (Fig. 10b).

### Aeromagnetic data

**Fig. 11:**
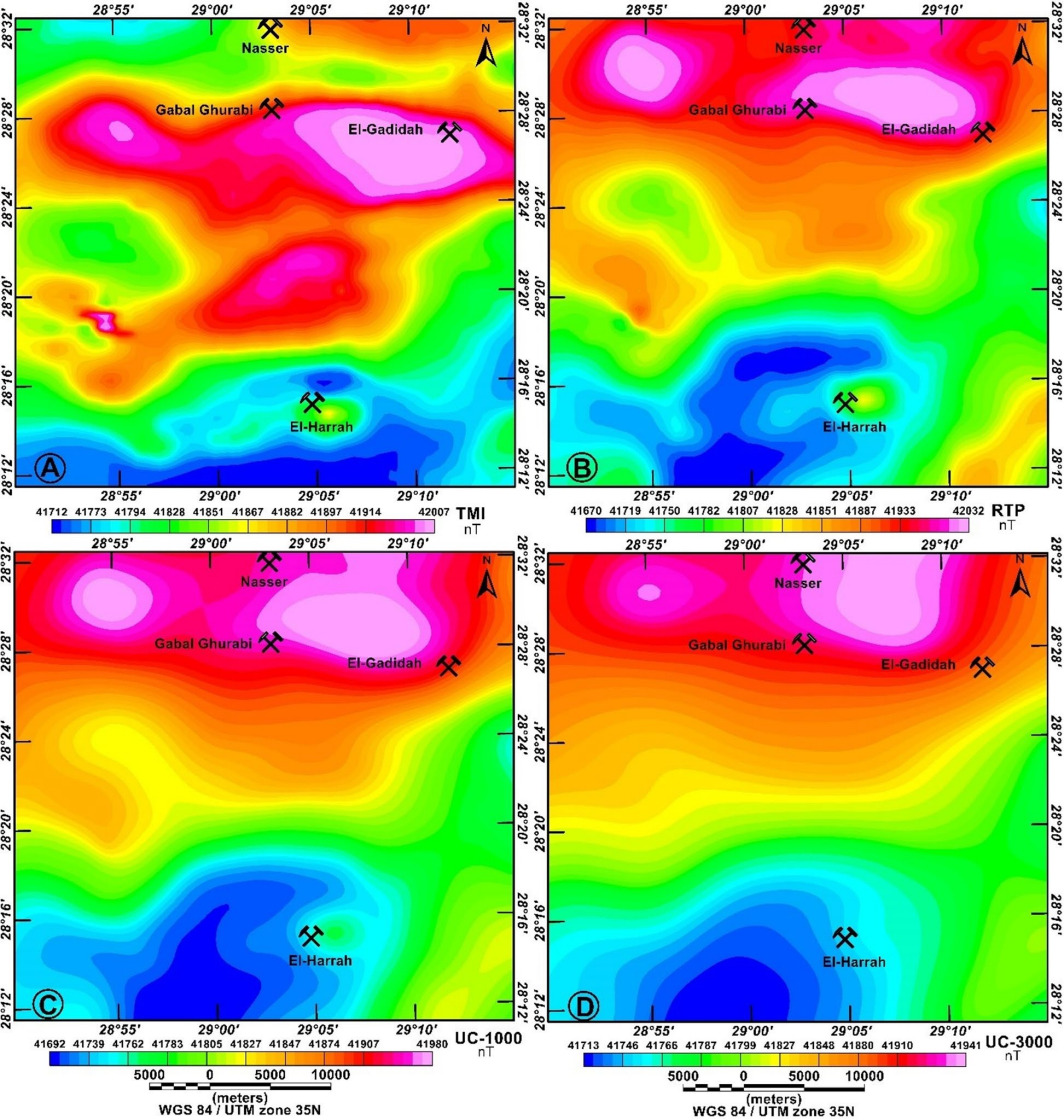
(**a**) TMI map; (**b**) RTP map; (**c**) UC-RTP at 1 km; (**d**) UC-RTP at 3 km of the study area. The figure was generated using Geosoft Oasis montaj (v8.3.3) software (Seequent; https://www.seequent.com/products-solutions/oasis-montaj/).

The RTP map of the studied region highlights that the magnetic anomalies identified earlier in the TMI map have shifted noticeably toward the north (Fig. 11a). This apparent shift arises because corrections for the Earth’s magnetic field inclination and declination reposition these anomalies to their actual geographic positions. The RTP map effectively demonstrates how variations in magnetic susceptibility among rock units, together with different geological structures, collectively influence the magnetic signatures. These signals reflect the interplay between subsurface composition and structural features at varying depths, producing distinctive magnetic responses.

The RTP map displays magnetic anomaly values ranging from 41,670 to 42,032 nT. These variations correspond to differing magnetic properties of the underlying rock materials. Two primary magnetic zones were delineated based on variations in magnetic intensity. The northern and northwestern sectors, along with a small portion in the southern area, are characterized by positive amplitude anomalies. These features range from ovoid to elongated in shape and generally trend NW–SE and E–W (Fig. 11b). They correspond closely to known iron ore mining zones, suggesting strong magnetization from ferromagnetic minerals. Conversely, the southern part of the map exhibits a broad, low-magnetic anomaly of irregular shape (Fig. 11b), implying the presence of less magnetic lithologies.

Figures 11c, d illustrate the upward continuation of the RTP map at altitudes of 1 and 3 km. This process is carried out to gain insights into the depth and orientation of basement structures. The upward continuation corresponds to depths of 0.5 and 1,5 km, respectively. This relation follows the general rule that the depth of data upward-continued to a height of z is approximately half of that value^86^. The high positive magnetic anomalies in the northern and northwestern regions retain nearly constant magnitudes up to 3 km, indicating their association with deep-seated basement rocks. Similarly, the low magnetic anomaly in the southern zone maintains its magnitude through the same upward continuation levels. In contrast, distinct high and low anomalies observed in the southern and central parts of the RTP map vary in amplitude with increasing continuation height, signifying shallow magnetic sources. The upward continuation technique thus minimizes the influence of local disturbances from near-surface sources and enhances the visibility of regional structural trends, providing a clearer representation of the underlying magnetic framework^27^.

Interpreting the RTP and UC maps qualitatively can often be unclear and challenging. To address this, signal enhancement techniques need to be applied to increase the clarity and strengthen the interpretation. One such approach involves the use of edge detection techniques, which play a crucial role in mineral exploration by assisting in the identification of magnetic sources and geological structures, such as fractures, faults, and contacts, that impact the distribution of mineral deposits^19^. Two high-precision edge detection filters, the enhanced tilt angle of the Horizontal Gradient (THGED) and the modified Gudermannian function of total horizontal gradient (MGTHG), were applied to the RTP data and UC maps to accurately map the edges of both deep and shallow magnetic sources.

**Fig. 12:**
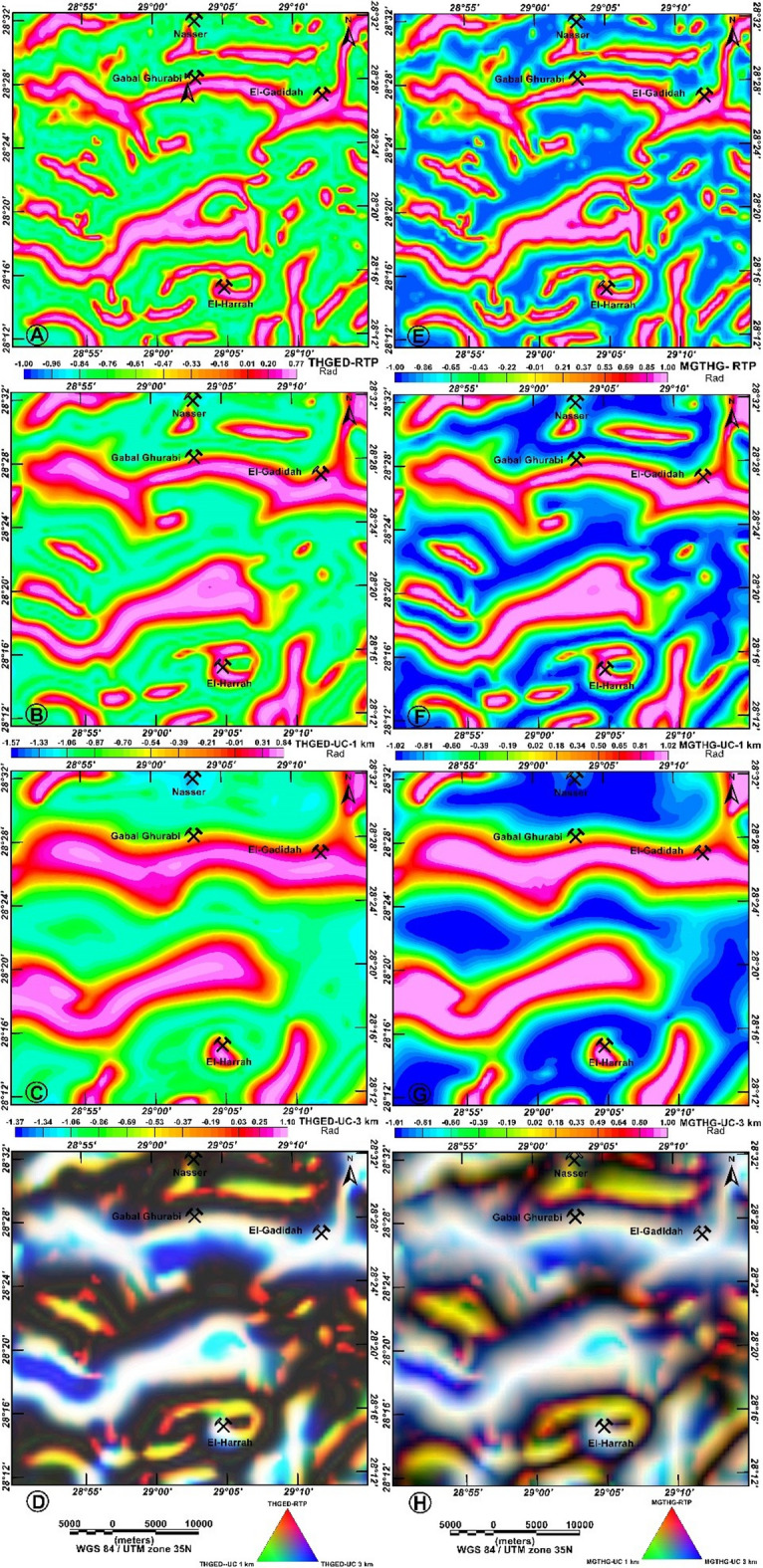
(**a**) THGED-RTP; (**b**) THGED-UC at 1 km; (**c**) THGED-UC at 3 km; (**d**) THGED-RTP, THGED-UC at 1 km, THGED-UC at 3 km. RGB ternary image; (**e**) MGTHG-RTP; (**f**) MGTHG–UC at 1 km; (**g**) MGTHG-UC at 3 km; (**h**) MGTHG-RTP, MGTHG-UC at 1 km, MGTHG-UC at 3 km. RGB ternary image of the study area. The figure was generated using Geosoft Oasis montaj (v8.3.3) software (Seequent; https://www.seequent.com/products-solutions/oasis-montaj/).

These high-precision filters demonstrated superior performance compared to conventional approaches by providing higher spatial resolution and enabling clearer differentiation between strong and weak magnetic sources. This improvement is especially evident in the southern part of the study area, where the conventional RTP map failed to distinguish subtle structural variations (Fig. 11a). Figure 12 presents the borders of magnetic sources identified using the THGED and MGTHG filters. These cutting-edge methods yield detailed and reliable results, enabling a more comprehensive understanding of the structural framework. When these filters were applied to UC data at altitudes of 1 km and 3 km (Figs. 12b, c, f, g), the number of detected anomalies decreased significantly, indicating that the majority of the features delineated in the THGED-RTP and MGTHG-RTP maps are shallow in origin.

**Fig. 13:**
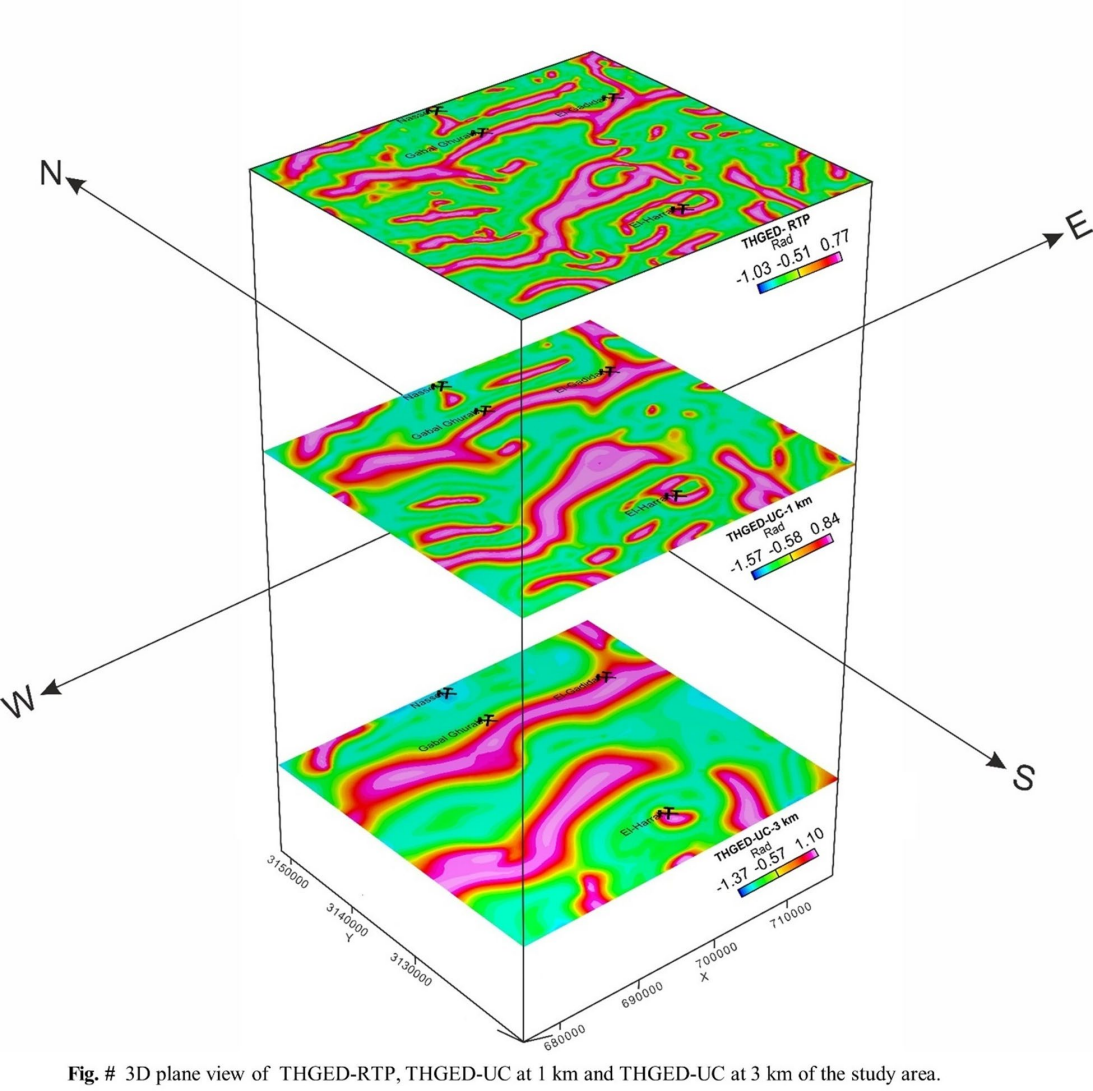
3D plane view of THGED-RTP, THGED-UC at 1 km, and THGED-UC at 3 km of the study area. The figure was generated using Geosoft Oasis montaj (v8.3.3) software (Seequent; https://www.seequent.com/products-solutions/oasis-montaj/).

**Fig. 14:**
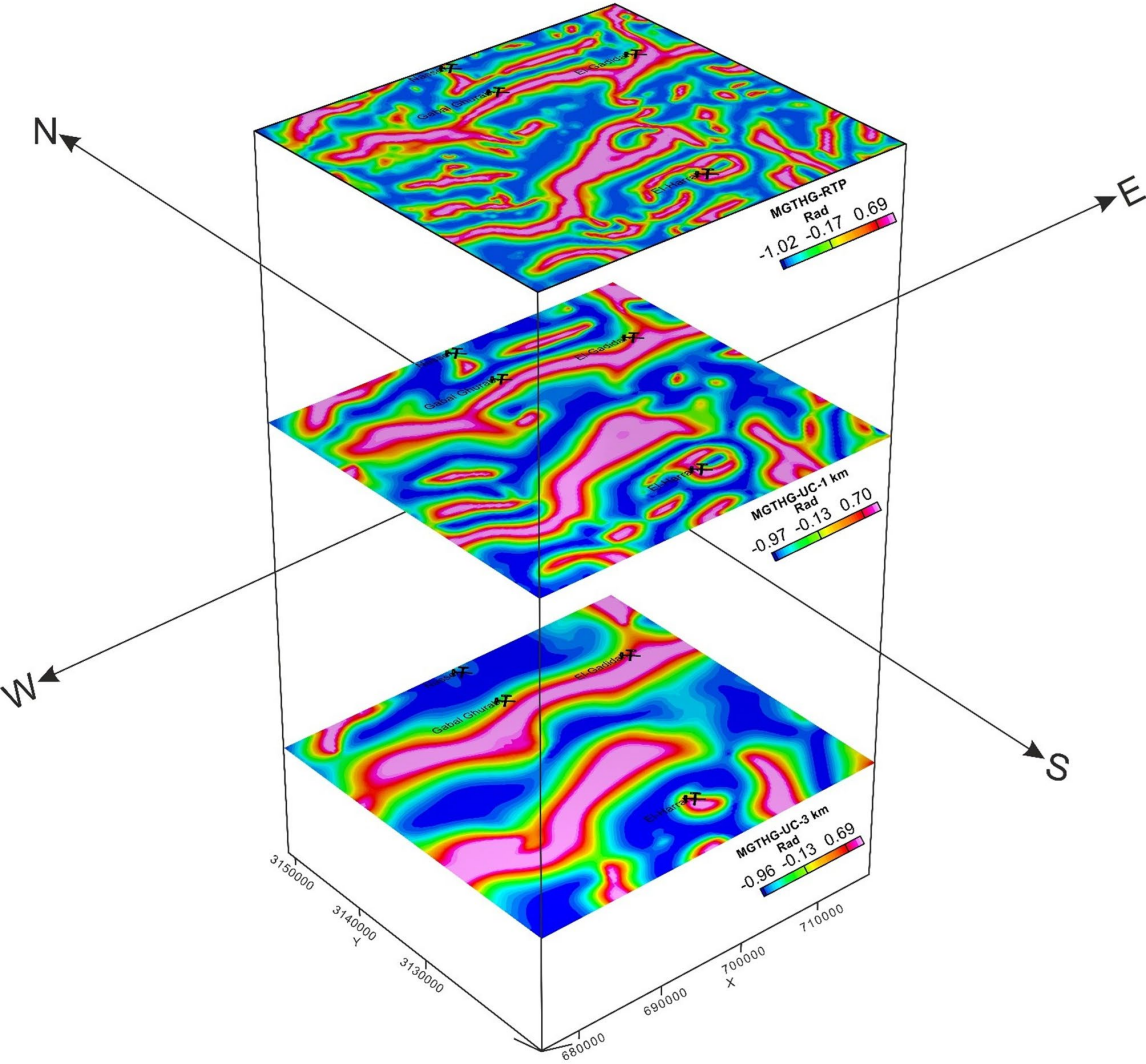
3D plane view of MGTHG-RTP, MGTHG-UC at 1 km, and MGTHG-UC at 3 km of the study area. The figure was generated using Geosoft Oasis montaj (v8.3.3) software (Seequent; https://www.seequent.com/products-solutions/oasis-montaj/).

The integration of the two filtering approaches markedly improved the reliability and consistency of magnetic interpretations. The resulting maps display positive magnetic trends predominantly oriented NE–SW and NW–SE, with subordinate N–S and E–W directions (Fig. 12). To visualize depth continuity of these structural lineaments, the outputs from three different THGED and MGTHG filtering steps were combined into an RGB composite (Figs. 12d, h), following the approach of Lino et al^87^.. This technique effectively discriminates between major and minor lineament sets based on their wavelength characteristics. Furthermore, Figs. 13 and 14 illustrate 3D plan-view representations of THGED and MGTHG results across multiple altitudes, highlighting how magnetic intensity and structure vary with depth.

**Fig. 15:**
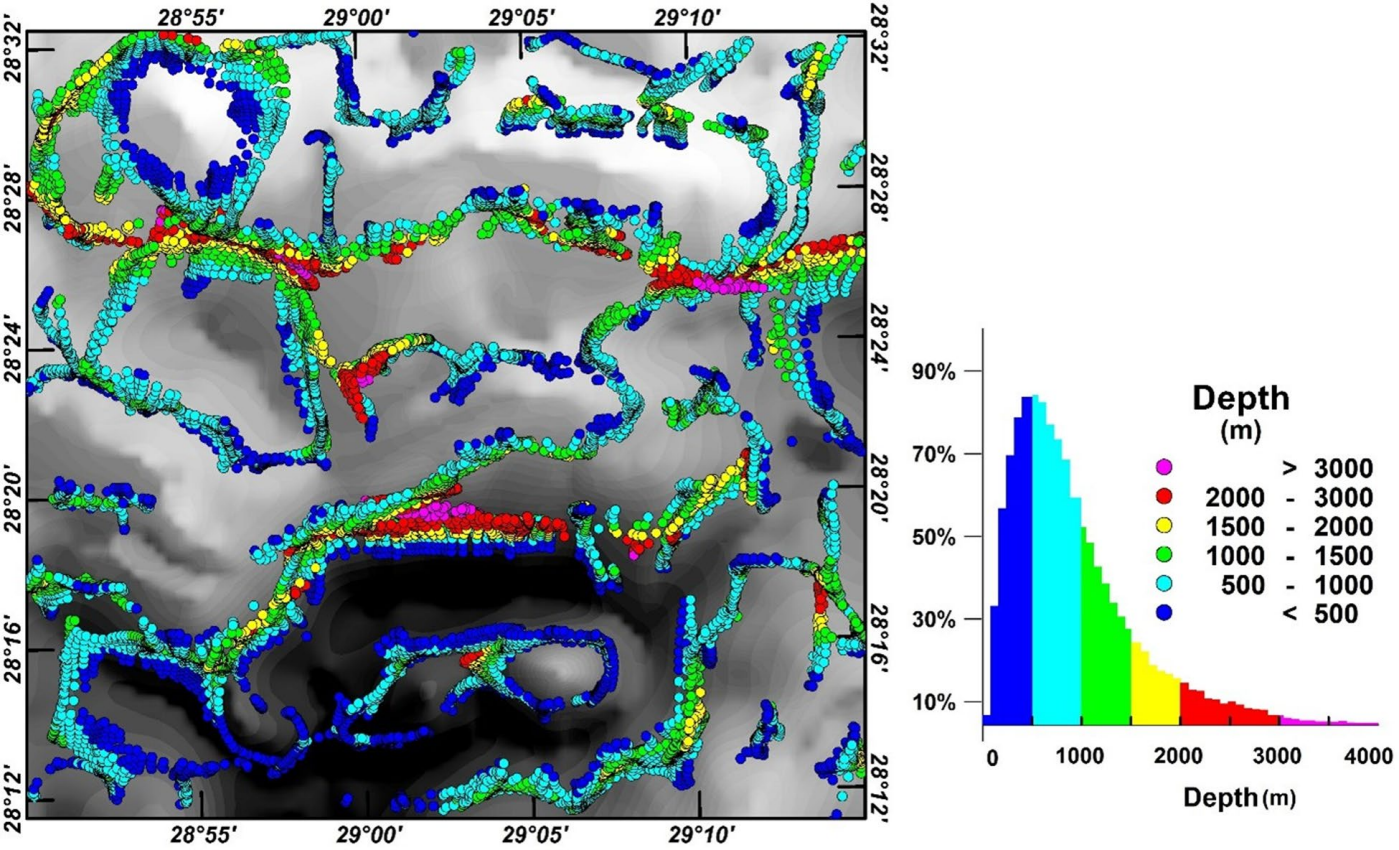
EUD depth solutions of the study area. The figure was generated using Geosoft Oasis montaj (v8.3.3) software (Seequent; https://www.seequent.com/products-solutions/oasis-montaj/).

**Fig. 16:**
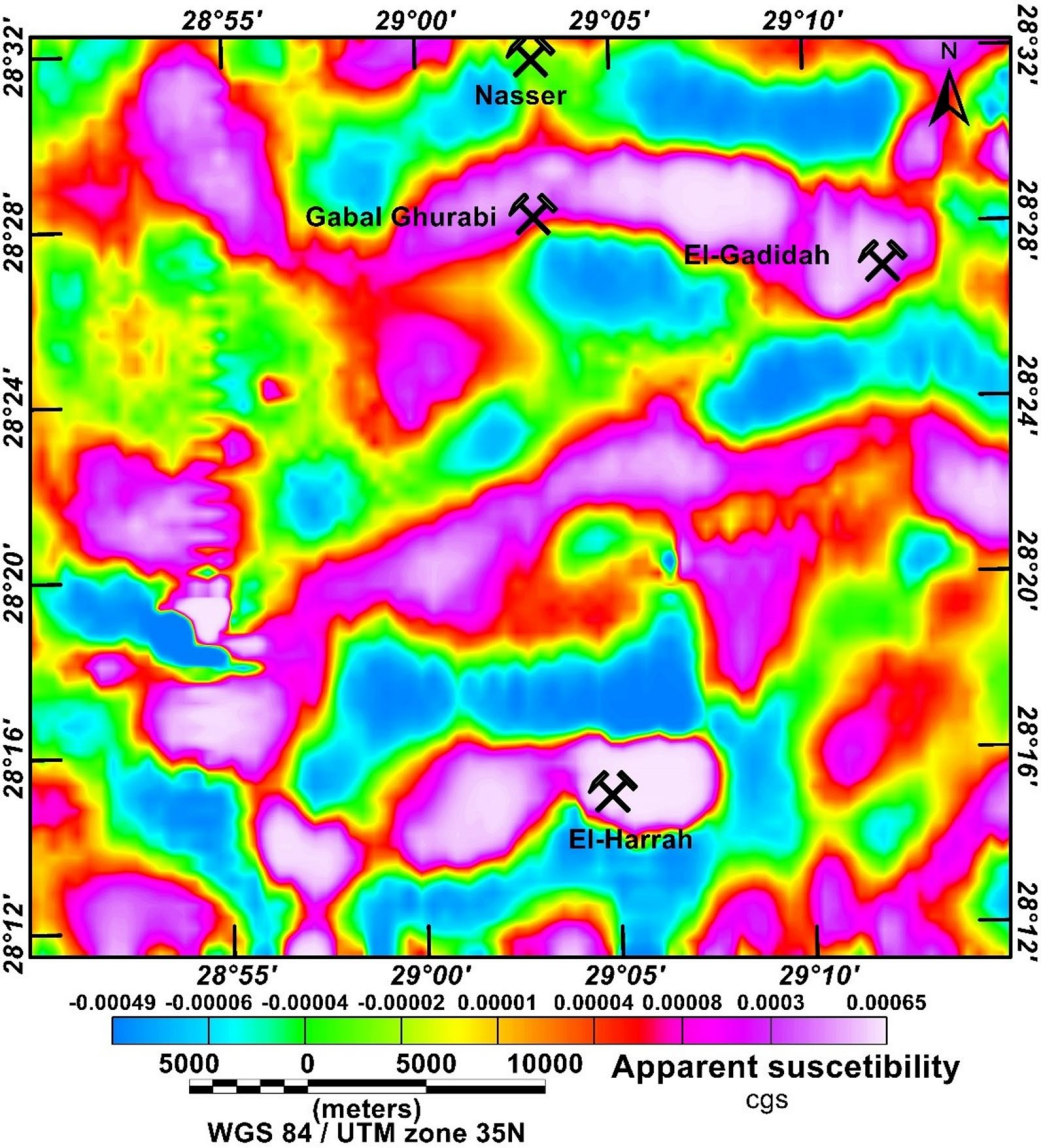
Apparent magnetic susceptibility (AMS) map of the study area. The figure was generated using Geosoft Oasis montaj (v8.3.3) software (Seequent; https://www.seequent.com/products-solutions/oasis-montaj/).

The RTP magnetic grid was employed as input for EUD to estimate the depth to the magnetic basement. This method effectively reduced noise and enhanced the visibility of subsurface structural features, even at considerable depths within the study area. A structural index (SI) value of 0 was selected, corresponding to contact-type sources, and a window size of 10 × 10 cells was utilized to compute the depths of magnetic anomalies. The EUD solutions show a pronounced clustering of depth estimates primarily trending NE–SW and NW–SE, with subordinate N–S and E–W orientations (Fig. 15). These alignments correspond to the dominant regional structural trends inferred from the RTP and edge-detection analyses. The statistical evaluation of depth estimates reveals that most solutions are concentrated within the upper 2 km, suggesting the presence of shallow geological formations. However, a subset of solutions extends to depths exceeding 3 km, reflecting deeper-seated features that likely represent significant faults, dykes, or basement structures influencing the magnetic field signature. An apparent magnetic susceptibility (AMS) map (Fig. 16) was generated to visualize spatial variations in the magnetic properties of rocks across the study area. Two distinct magnetic susceptibility zones were identified. The high-susceptibility zones, ranging from 0.0003 to 0.00065 SI, are primarily located near known iron ore deposits, indicating strong magnetization responses. Conversely, the low-susceptibility zones, with values between − 0.00049 and − 0.00002 SI, correspond to areas largely devoid of magnetic minerals. These patterns delineate significant lithological contrasts and potential mineralization zones within the subsurface.

**Fig. 17:**
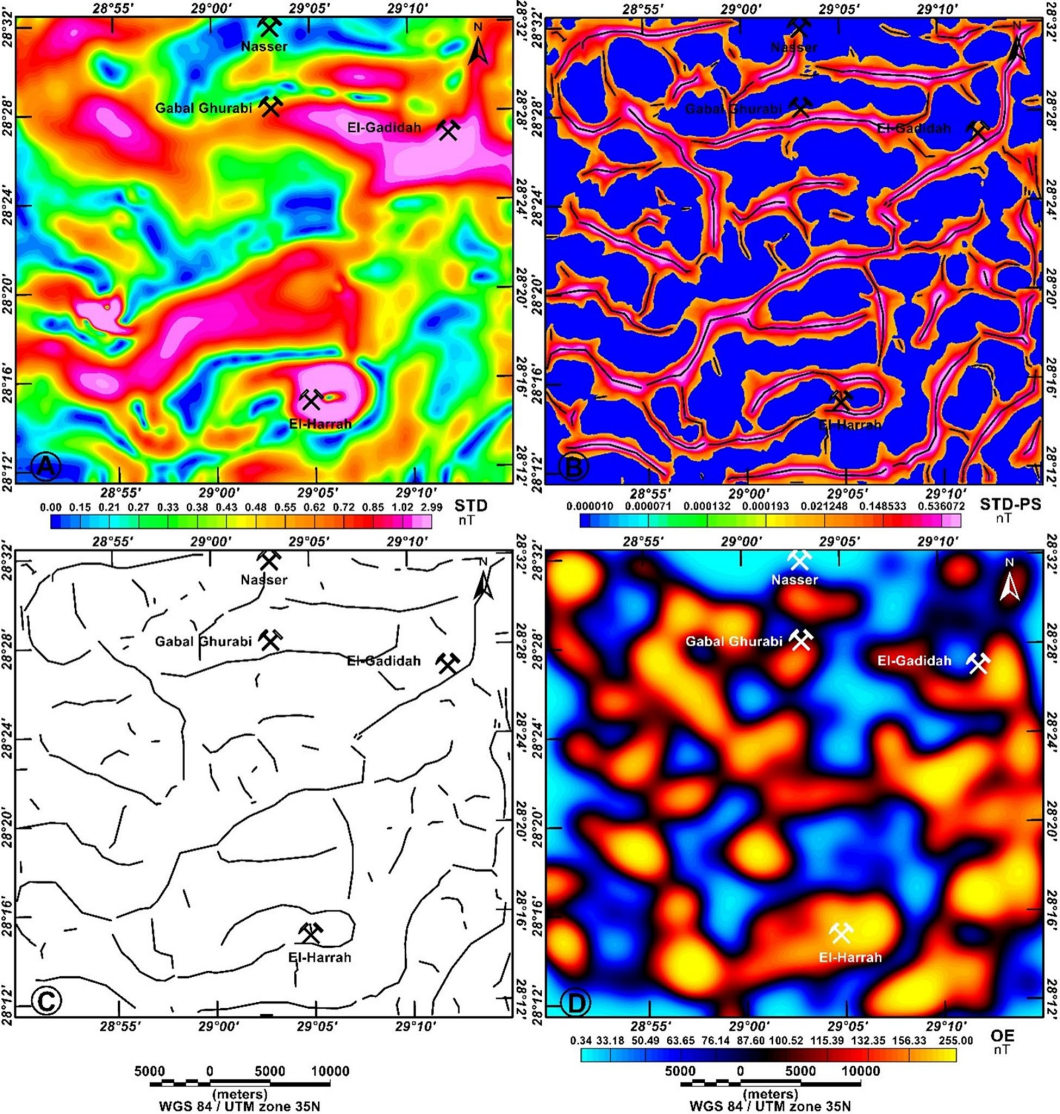
CET-GA results: (**A**) standard deviation, (**B**) phase symmetry, (**C**) generated automated lineaments, and (**D**) entropy heat map. The figure was generated using Geosoft Oasis Montaj (v8.3.3) software (Seequent; https://www.seequent.com/products-solutions/oasis-montaj/).

The CET-GA approach was employed to analyze image textures derived from the RTP magnetic dataset, effectively highlighting zones of structural complexity across the study area. As part of the workflow, the standard deviation (STD) was calculated to delineate areas with high textural variability, which typically indicate magnetic discontinuities (Fig. 17a). The STD output was then used to generate a Phase Symmetry (PS) map (Fig. 17b), enabling enhanced recognition of structural lineaments and zones of interest. This method improved the lateral separation of magnetic structures, thereby enhancing interpretive clarity. To ensure a comprehensive structural analysis, the study used all orientations at a small scale of 100 m, maximizing the detection of fine-scale linear features. Only positive features, such as persistent positive magnetic responses associated with dykes, were considered in this step. The identified linear features were subsequently extracted using skeletonization and vectorization techniques, which produced a detailed vectorized lineament map (Fig. 17c). The resulting vectorization map revealed dominant structural trends oriented NE–SW and NW–SE, with subordinate lineaments aligned N–S and E–W (Fig. 17c). These orientations coincide with the regional tectonic fabric. The Orientation Entropy map (Fig. 17d) further identified areas exhibiting high structural complexity, where multiple geological features intersect or show directional variability. Notably, these high-entropy zones coincide spatially with known mining sites, confirming their association with mineralized structures.

**Fig. 18:**
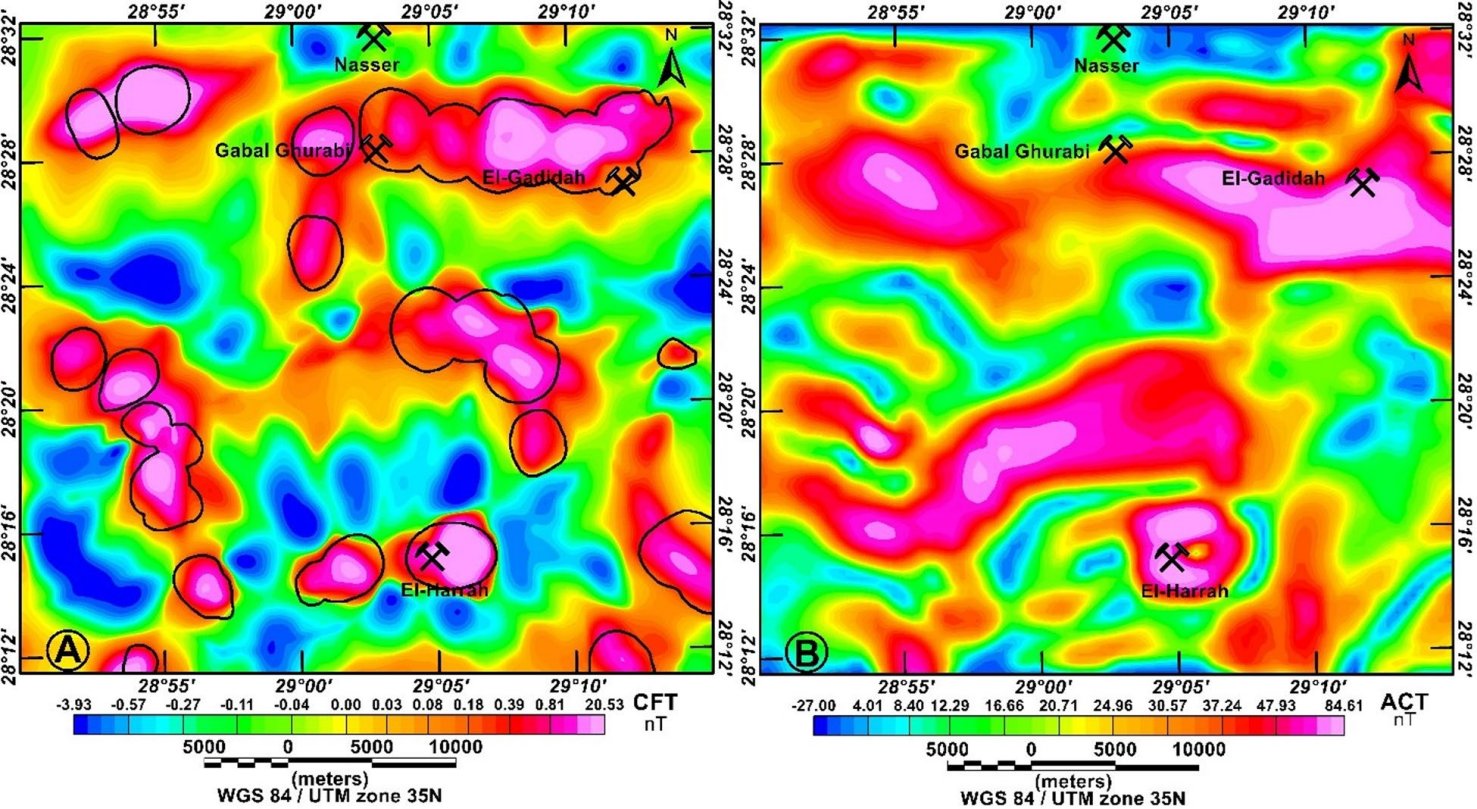
CET-PA results: (**A**) Circular Feature Transform (CFT), (**B**) Amplitude Contrast Transform (ACT) of the study area. The figure was generated using Geosoft Oasis Montaj (v8.3.3) software (Seequent; https://www.seequent.com/products-solutions/oasis-montaj/).

In the Supportive stage, algorithms from the CET-PA module were applied to the RTP data to detect circular and semi-circular magnetic patterns indicative of porphyry-type intrusions (Fig. 18). These features often correspond to hydrothermal alteration zones and potential sites for iron ore mineralization. The detected porphyritic intrusions show strong spatial correlation with the previously mapped structural complexity zones and share similar NE–SW and NW–SE orientations. Their circular or semi-circular geometries are characteristic of porphyritic intrusive bodies, making them valuable indicators in mineral exploration. The spatial distribution of porphyry-type features shows a pronounced concentration near known mining zones, with additional occurrences in the northwestern and central sectors, highlighting multiple economically prospective regions (Fig. 18). Comparison of the CET-PA results with outputs from RTP, THGED, MGTHG, EUD, Apparent Magnetic Susceptibility, and Orientation Entropy analyses demonstrates strong consistency, collectively delineating the most promising ore deposit targets and significantly reducing interpretational ambiguity.

### Geochemistry

The major oxide compositions of the analyzed samples from the El-Bahariya Oasis are presented in Table 2. The dataset reveals a wide compositional range reflecting carbonate, iron-rich, and siliceous lithologies. In this study, the silica content (SiO_2_) varies markedly from 1.40 to 88.06 wt%, indicating strong lithological heterogeneity. Carbonate-rich samples (BR1, BR8, BR9) are characterized by very low SiO₂ (< 7.1 wt%) and exceptionally high CaO contents (35.54–50.56 wt%), accompanied by high loss on ignition (35.4–45.97 wt%), consistent with calcite- to dolomite-dominated compositions (Table 2). In contrast, a group of samples (BR3, BR4, BR5, BR6, BR7) exhibits very high total iron contents, with FeOᵗ ranging from 25.99 to 46.05 wt%, defining ironstone to high-grade iron ore compositions. These samples show moderate SiO₂ (41.28–66.95 wt%) and relatively low CaO and MgO, reflecting the dominance of iron oxides (hematite ± goethite) over carbonate phases (Table 2). Al₂O₃ and TiO₂ contents are uniformly low across all samples (Al₂O₃ ≤1.77 wt%, TiO₂ ≤0.14 wt%), suggesting minimal detrital clay input and supporting a chemical to biochemical sedimentary origin for the iron-rich units. Conspicuously, P₂O₅ concentrations are elevated in the iron-rich samples (0.45–0.84 wt%), indicating the presence of apatite and a genetic link to phosphatic iron formations typical of the Bahariya district. One sample (BR13) is distinguished by a very high SiO₂ content (88.06 wt%) and negligible FeOᵗ, CaO, and MgO, corresponding to a siliceous lithology, likely chert or strongly silicified sandstone, associated with the iron-bearing sequence. Manganese exhibits localized enrichment, with MnO reaching 4.85 wt% in BR9, indicating the presence of Mn-bearing carbonate phases and suggesting variable redox conditions during deposition or diagenesis.

**Table 2:** Major oxides (wt%) analysis of the collected samples from El-Bahariya, Western Desert, Egypt.

Rock type	Iron stones					Carbonatized rocks			Silicic rock
Sample No.	**BR3**	**BR4**	**BR5**	**BR6**	**BR7**	**BR1**	**BR8**	**BR9**	**BR13**
SiO_2_	66.95	54.86	49.92	50.73	41.28	7.07	1.4	1.44	88.06
TiO_2_	0.06	0.08	0.14	0.09	0.12	0.12	0.02	0.03	0.02
Al_2_O_3_	1.2	0.9	1.77	1.16	0.64	1.44	0.5	0.5	0.24
FeO^t^	25.99	33.63	33.51	28.52	46.05	4.43	0.36	0.26	0.41
MnO	0.05	0.11	0.1	0.09	0.05	7.76	0.02	4.85	0.01
MgO	0.13	0.28	0.77	0.57	0.3	0.92	15.48	0.46	0.08
CaO	0.91	2.12	5.46	3.7	1.74	37.55	35.54	50.56	6.15
Na_2_O	0.15	0.07	0.17	1.95	0.05	1.6	0.12	0.06	0.09
K_2_O	0.04	0.03	0.08	0.09	0.11	0.14	0.03	0.05	0.03
P_2_O_5_	0.48	0.69	0.45	0.47	0.84	0.02	0.16	0.04	0.16
SO_3_	0.17	1.84	0.96	3.63	2.91	0.6	0.33	0.86	0.42
Cl	0.13	0.05	0.24	1.88	0.04	1.38	0.05	0.04	0.08
LOI	3.46	5.02	6.21	6.84	5.62	35.4	45.97	39.92	4.2
Sc	4.4	16.8	16.1	11.3	12.1	42.3	21.4	38.5	5.5
V	492.9	846	227.2	445.8	15	373	5.2	44.5	10.6
Cr	222.1	435	45.7	130.7	129	26.9	9.4	8	10.5
Co	91.5	244.2	95	93.9	N.D	57.4	1.1	26.7	1.1
Ni	225.7	456.5	98.2	130.4	47.2	188	5.5	146.2	6.4
Cu	17.8	31.4	12.4	12.2	17.4	9.9	4.6	9.2	5.1
Zn	149	311.4	84.7	86.6	98.6	49.9	26.4	40	21.4
Ga	1.6	3	N.D	N.D	N.D	N.D	0.5	N.D	1.4
As	23.3	38.9	66.9	79.9	5.5	15.5	N.D	0.3	N.D
Se	0.3	3.2	1.2	1	2.1	1.3	N.D	0.8	N.D
Br	2.4	4.2	2.8	3.8	7.9	1.7	0.5	N.D	0.9
Rb	0.7	0.7	1.3	2.7	2.2	1.2	0.5	N.D	0.5
Sr	24.7	58.6	102.8	242.9	204,9	305.5	119.1	218	40.4
Y	19	18.3	17.3	16.4	6.3	20	3.7	35.2	2.2
Zr	68.8	95.4	201.3	197.3	241.2	148.5	8.3	10.6	3.5
Nb	2.2	2.6	2.3	1.1	3.2	1.5	0.1	0.4	0
Mo	4.7	3.5	4.1	2.8	2.4	4.3	0.8	2.5	0.2
Ag	0.1	0.2	N.D	0.8	0.4	0.6	N.D	0.3	0.4
Cd	0.1	N.D	0.9	1.5	0.6	1.8	N.D	9.9	1.1
Sn	N.D	N.D	1.6	2.2	2.5	15.9	N.D	23	1
Sb	N.D	N.D	5.8	3.1	0.6	2.7	N.D	4.7	2.4
Te	N.D	N.D	N.D	N.D	N.D	N.D	2.1	N.D	7
I	15.8	18.3	31	19.9	14.5	44.5	9	19.8	3.9
Cs	35.4	42.4	54.3	44.2	34.9	2.9	13.5	21.1	5.2
Ba	78.4	191.4	413.5	145.6	5234.7	9935	36.3	5317.6	60.9
La	N.D	6.2	N.D	7.1	2.1	22.3	7.2	42.3	8.9
Ce	4.2	N.D	20.9	N.D	19.2	N.D	N.D	N.D	N.D
Nd	N.D	26.1	0.5	N.D	N.D	N.D	1.4	2.8	N.D
Sm	1.5	N.D	16	N.D	N.D	16.2	N.D	14.6	0.3
Hf	N.D	N.D	N.D	N.D	N.D	N.D	0.1	N.D	2.3
Ta	7.5	N.D	0.1	N.D	N.D	0.7	0.6	0.2	1.9
W	136.3	281.7	204	168.4	387.3	16.5	3.3	N.D	1.3
Hg	N.D	N.D	N.D	N.D	N.D	N.D	N.D	8.6	N.D
Tl	3.6	7.6	9.1	3.2	15.5	10.8	2.4	8.8	2.4
Pb	2.3	1.2	2.4	2.4	3.3	2.9	N.D	1.7	1
Bi	N.D	2.9	2.5	1.5	2.9	2.3	N.D	N.D	N.D
Th	6	3.3	1.1	3.3	1.9	4.5	0.1	1.5	1.2
U	1.9	1.2	2.1	1.2	N.D	0.4	1.9	N.D	2.2

N.D. Under detection limit.

Whole-rock major oxide analysis (Table 2) reveals three chemically and mineralogically distinct endmember compositions within the El-Bahariya sequence. Iron-rich low-Mn samples (BR3–BR7) are characterized by high total iron (FeOt = 25.99–46.05 wt%), with sample BR7 achieving 46.05 wt%.% FeOt, approaching the industrial-grade threshold of 47.6 wt% Fe reported for existing El-Gedida mine ore. These samples display moderately elevated silica (SiO₂ = 41–67 wt%) and characteristically low calcium and magnesium (CaO < 6 wt%, MgO < 1 wt%), with negligible alkali oxides (Na₂O + K₂O < 2 wt%) and very low alumina (Al₂O₃ <2 wt%). Loss on ignition (LOI = 3.5–6.2 wt%) is modest, consistent with oxidized iron-bearing phases. The uniformly low Al₂O₃ and TiO₂ (typically < 0.15 wt%) indicate minimal detrital silicate input, excluding a simple clastic weathering origin and instead pointing to chemogenic (hydrogenous and/or hydrothermal) precipitation.

Carbonate-hosted samples (BR1, BR8, BR9) constitute a chemically distinct group with very high CaO (35–50 wt%) or MgO (up to 15.48 wt% in BR8), high loss on ignition (35–46 wt%), and crucially, very low total iron (FeOt < 5 wt%). Two of these samples (BR1, BR9) display anomalously high manganese (MnO = 7.76 and 4.85 wt%, respectively), approximately 80–150 times higher than low-Mn ironstones. Silica content is correspondingly low (SiO₂ = 1–7 wt%), and aluminum remains low (Al₂O₃ < 1.5 wt%), confirming that these are dominantly carbonate with minimal detrital silicate contamination. The elevated LOI is consistent with the breakdown of carbonates (calcite and/or dolomite) during heating. Phosphorus pentoxide (P₂O₅) shows systematic variation: iron-rich samples contain 0.45–0.84 wt% P₂O₅, markedly higher than carbonate samples (0.02–0.16 wt%) and the siliceous sample (0.16 wt%), pointing to the presence of apatite in iron ores and consistent with syndepositional or early-diagenetic phosphate incorporation. Sulfur (SO₃) is variable but notable in certain samples: carbonate and siliceous samples show low SO₃ (< 1 wt%), while some ironstones (BR4, BR6, BR7) reach 1.8–3.6 wt% SO₃, likely reflecting sulfate phases (e.g., barite, jarosite) or relict sulfide minerals altered to sulfate. Siliceous sample BR13 is compositionally isolated, with SiO₂ = 88.06 wt%, negligible iron and manganese, and trace amounts of other oxides, representing barren quartzite or silica-cemented sandstone.

**Fig. 19:**
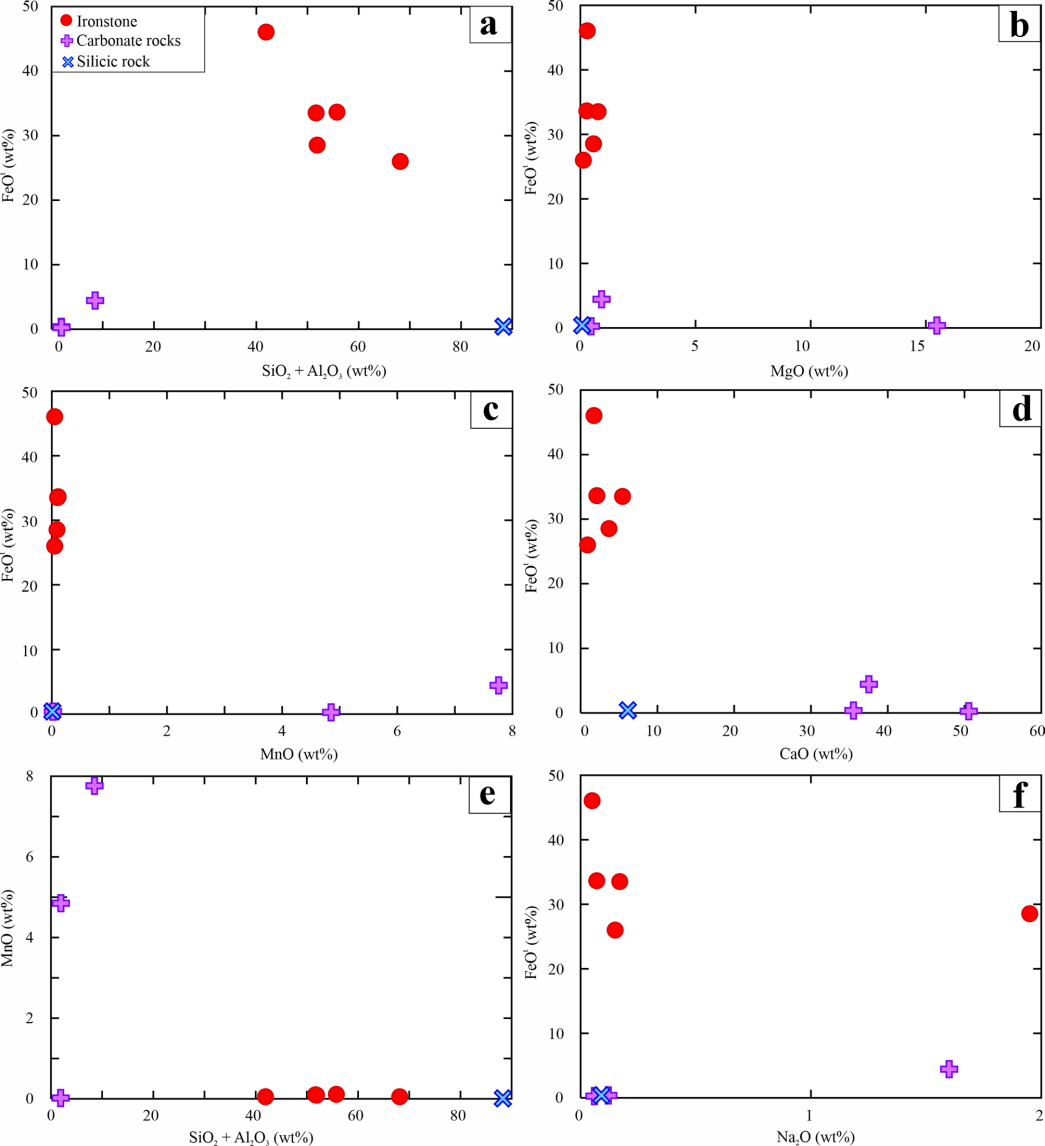
Harker diagrams showing geochemical discrimination of three lithofacies groups. Group 1 (Iron stones) economic low-Mn ironstones (FeO^t^ = 26–46 wt%, MnO < 0.11 wt%), Group 2 (Carbonate rocks) non-economic high-Mn carbonates (MnO 4.85–7.76 wt%), and Group 3 barren siliceous rocks.

Trace element patterns (Table 2) sharply differentiate the three lithofacies groups, providing insights into ore-formation processes. Iron-rich samples (BR3–BR7) are dramatically enriched in elements classically associated with chemical sedimentation and hydrothermal systems: barium (Ba = 5–413 ppm), vanadium (V = 15–846 ppm, with BR4 notably enriched at 846 ppm), chromium (Cr = 8–435 ppm), nickel (Ni = 5.5–456.5 ppm), zinc (Zn = 85–311 ppm), and tungsten (W = 1.3–387 ppm). Vanadium and tungsten exhibit a positive correlation with FeOt, with the highest concentrations observed in mid-grade samples, suggesting an association with magnetite or other redox-sensitive iron phases. Sr is elevated relative to clastic standards (24.7–242.9 ppm), consistent with incorporation into iron-oxide minerals or residual sulfate phases. Arsenic (As) reaches 23–80 ppm in selected samples (BR5, BR6), suggesting minor arsenate minerals or arsenic incorporated during hydrothermal mixing or supergene alteration. Carbonate-hosted samples (BR1, BR9) show a strikingly different trace element signature, most notably extreme barium enrichment: BR1 contains 9935 ppm Ba, and BR9 contains 5318 ppm Ba up to 25 times higher than even the most Ba-rich ironstones. This anomalous Ba enrichment reflects the precipitation of abundant barite (BaSO₄), a mineral indicator of hydrothermal fluid–seawater mixing and associated sulfate reduction in reducing environments. Manganese-rich carbonate samples also show elevated Sr (219–305 ppm) and moderate concentrations of redox-sensitive metals (V = 5–373 ppm, Cr = 9–27 ppm, Ni = 5.5–188 ppm). The trace element partitioning, specifically, the decoupling of Ba and Mn enrichment from Fe enrichment, is consistent with precipitation at redox boundaries under reducing conditions, where Mn and Ba preferentially concentrate while Fe oxides remain stable in adjacent, more oxidizing horizons. Siliceous sample BR13 is depleted in virtually all trace elements, consistent with silica dilution of any chemical signal.

Harker diagrams (Fig. 19) identify three distinct lithofacies with clear compositional boundaries. Iron-rich, low-Mn ironstones (BR3–BR7) exhibit high FeOt (26–46 wt%), moderate SiO₂ (41–67 wt%), low MnO (< 0.11 wt%), and depleted Al₂O₃/TiO₂ ratios (< 2 and < 0.15 wt%), suggesting a chemogenic origin rather than clastic (Table 2). The inverse correlation between FeO^t^ and SiO₂-Al₂O₃ (Fig. 19a) indicates minimal detrital silicate content. Notably, the Fe–Mn diagram (Fig. 19c) shows a clear separation: low-Mn ores (MnO < 0.15 wt%) versus carbonate-hosted samples (BR1, BR9) with MnO levels of 5–8 wt%, reflecting redox-controlled partitioning. The inverse relationship between Fe and CaO (Fig. 19d) demonstrates calcium’s exclusion from iron oxides and its preferential incorporation into carbonates (CaO 35–50 wt%; Table 2). Phosphorus enrichment (P₂O₅ = 0.45–0.84 wt%) suggests syndepositional apatite of marine origin. Mg tends to partition into dolomite-rich samples (MgO up to 15.48 wt%, Fig. 19b), while alkali oxides remain low (Na₂O + K₂O < 2 wt%, Fig. 19f), indicating feldspar weathering is absent.

## Discussion

### Machine learning lithofacies classification

Machine learning algorithms (Random Forest, Support Vector Machines) applied to the ASTER + Sentinel-2 dataset significantly improve lithological classification accuracy and repeatability compared to visiual image interpretation or unsupervised spectral clustering. Compared with previous remote-sensing studies of the Bahariya district, which typically relied on band-ratio and PCA techniques applied to a single multispectral sensor, the present multi-sensor and machine-learning workflow delivers higher classification accuracy, sharper lithofacies boundaries, and quantitative discrimination of ore-grade horizons from non-economic ferruginous and carbonate units.

Random Forest, in particular, excels at multispectral lithofacies discrimination by learning non-linear relationships between reflectance spectra and training data (field-sampled rock types), achieving an overall accuracy (OA) of 91.73% (Table 1). The results demonstrate the efficacy of these algorithms in accurately mapping lithological units, validated through field observations and geochemical analyses (Fig. 2; Table 2). Both classifiers utilized ASTER (9 bands) and Sentinel-2 (11 bands) reflectance spectra at the exact field sample coordinates, with 80% of the data used for training and 20% for validation. RF outperformed SVM, achieving overall accuracy (OA) of 91.73% and Kappa coefficient of 89.99%, with the highest F1-scores for low-Mn iron-rich lithofacies (0.89) and the lowest for rare barren siliceous rocks (0.72) (Table 1; Fig. 4). The RF classification map (Fig. 4d) delineates low-Mn ore-grade zones (magenta and red pixels, 186.3 km²) that spatially coincide with PRISMA ferric iron index anomalies (Fig. 7a), SAM hematite/limonite detections (21.51 km² hematite + 5.29 km² limonite, Fig. 9), and aeromagnetic susceptibility highs (Fig. 18), validating RF lithofacies discrimination accuracy and confirming that machine-learned ore-zone boundaries are geologically realistic and exploration-ready. Key advantages of RF over SVM include: (1) sharper spatial coherence in the classified map (Fig. 4d vs. 4c), reducing spectral salt-and-pepper noise; (2) superior discrimination of the Bahariya Formation; (3) fewer spectral artifacts in contact zones; and (4) overall higher global accuracy metrics. The principal trade-off is a marginally lower recall for Iron Ore Deposits (80.18% vs. 85.79% for SVM; Table 1), resulting in approximately 5.6% points more undetected ore pixels at the deposit scale. This recall reduction is, however, accompanied by a higher precision (94.93% vs. 93.09%), which means that the RF map contains fewer false-positive ore pixels and is therefore more reliable for prioritizing drill-ready targets. The geological interpretation of this error budget is that the residual ore-pixel errors correspond predominantly to thin, gradational ore margins that grade into the surrounding ferruginous Bahariya and carbonate-hosted Qazzun formations, where the multispectral reflectance contrast is intrinsically low. Such pixels are best recovered by the complementary hyperspectral PRISMA SAM and ferric-iron index analyses (Figs. 7 and 9), which are sensitive to subtle hematite/goethite absorption features at 0.6, 0.9, and 1.04 μm and which directly capture the mineralogical transitions that the multispectral RF classifier cannot fully resolve. However, RF’s higher spatial continuity and overall classification accuracy justify its selection as the primary lithological map for exploration targeting, with SVM results considered as an ensemble validation layer for high-confidence ore identification.

### Multisource datasets for iron deposits mapping

Hyperspectral PRISMA-derived mineral indices and multispectral machine learning classification jointly delineate previously unmapped, high-potential mineralization zones within the southern and central sectors of the El-Bahariya depression, extending beyond the footprints of known economic deposits (Nasser, Ghorabi, El-Gadidah, El-Harrah). These newly identified zones exhibit spatial extents and spectral characteristics consistent with three key datasets: (1) low-Mn, iron-rich lithologies identified by Random Forest classification (Fig. 4d; OA = 91.73%, Kappa = 89.99%), (2) positive magnetic susceptibility anomalies revealed by aeromagnetic total magnetic intensity and residual intensity grids processed via edge detection (Tilt angle, Total Horizontal Gradient) and advanced spectral analysis techniques (Continuous Energy Texture Gradient Amplitude, CET-GA), and (3) geochemically-defined low-Mn ore endmembers characterized by FeOt = 26–46 wt%, MnO < 0.11 wt%, and diagnostic Harker diagram clustering (Table 4; Group 1 classification). The remarkable spectral, spatial, and geochemical coherence among these three independent data streams validates the integrated targeting approach and confirms that new ore-grade iron oxide deposits are present in under-explored depression sectors.

Spectral signatures from hyperspectral PRISMA analysis provide the foundation for recognizing ore bodies. The ferric iron index (b33/b21), targeting hematite and goethite absorption features at 0.9 and 1.04 μm, respectively, delineates Fe³⁺-bearing mineral assemblages (green pixels, Fig. 8a) concentrated in known mines and distributed throughout the northeastern plateau. The ferrugination composite index ((b132/b33) + (b33/b21)) integrates ferric iron and iron-hydroxide signatures across the 0.6, 0.9, and 2.2 μm spectral windows (yellow zones, Fig. 8e), revealing zones of intense iron oxide precipitation and oxidation-state variation reflecting both primary hydrothermal and secondary supergene contributions. These spectral signatures are not random noise: they correspond precisely to high-intensity pixels in the RF-classified iron ore deposits (magenta/purple zones, Fig. 4d), indicating that machine learning successfully captured the hyperspectral mineralogy in coarser multispectral bands (ASTER and Sentinel-2). Furthermore, the spatial overlap between PRISMA mineral index anomalies and geochemically-defined Group 1 (low-Mn economic ore) targets suggests that spectral detection of ore-grade iron oxides is a reliable, cost-effective, and repeatable exploration tool.

Magnetic anomaly interpretation enables the identification of magnetite-bearing basement rocks, the depth of the basement, and concealed fault/fracture systems that control ore localization^88^. Although the survey was acquired in 1984, it remains suitable for regional interpretation because the main anomaly patterns reflect geologically stable basement structures. The survey was originally designed to image basement structure at an exploration scale, and the magnetic anomalies we interpret are primarily related to basement rocks and structural features, which are effectively time-invariant over the decades since acquisition^59^. Nevertheless, we recognize that post-acquisition land-use and infrastructural development may have introduced additional near-surface cultural noise, predominantly affecting the short-wavelength components of the field. To minimize the influence of such effects, multiple gridding algorithms were evaluated, and the processed results were iteratively compared with the geological framework and other geophysical constraints. Within this framework, the main objectives of the aeromagnetic analysis are to estimate basement depth, delineate structural features within the basement rocks, and detect subsurface structural features that control ore localization.Upward continuation (UC) and reduced-to-pole (RTP) filtering isolate deep, magnetite-rich basement sources in the northern and northwestern El-Bahariya depression (magnetic highs, Fig. 11) from shallow sedimentary cover and altered zones in the south (magnetic lows, Fig. 11), demonstrating the spatial separation of primary ore-hosting basement from overlying non-magnetic strata^27^. The attenuation of short-wavelength anomalies with increasing height validates the method’s effectiveness in isolating regional magnetic components and reducing noise from shallow sources. THGED and MGTHG edge-detection filters sharpen anomaly gradients and precisely delineate magnetic boundaries, faults, and contacts that are obscured in traditional RTP/UC maps (Fig. 12), thereby enhancing the structural definition essential for mineral targeting^19,58,59^. The combined UC, RTP, and edge-detection approach provides comprehensive subsurface structural and lithological control on the magnetic field^27^.

The observed NE–SW and NW–SE magnetic trends (Figs. 11 and 12) are consistent with regional basement fault systems documented in the El-Bahariya structural framework^31,77,89–91^, indicating that these faults have controlled both structural deformation and mineralization processes. Euler Deconvolution (EUD) depth estimates cluster along NE–SW and NW–SE trends, with predominantly shallow solutions (< 2 km) indicating uplifted, structurally high basement throughout most of the depression, while fewer deeper solutions (> 3 km) at fault intersections mark presumed intrusive bodies or deep structural features serving as conduits for mineralizing fluids (Figs. 15, 77, 89 and 92–94]. The clustering of EUD solutions spatially matches field-documented brittle and ductile structures (fractures, folds, fault-filled veins) in glauconitic and ore-bearing strata (Figs. 1 and 3e and f), confirming the propagation of basement-lineaments through the sedimentary cover and their direct structural control on ore-body geometry upward. The structural index was selected according to the inferred source geometry rather than the composition of the mineralized body^69–71^. An SI value of 0 was used because the observed magnetic anomalies are interpreted to be dominated by contact-type sources, including lithologic boundaries, fault-filled vein zones, and contact-related discontinuities^69–71^. In the present sedimentary setting, the iron oxide mineralization is considered structurally and stratigraphically controlled, so that its magnetic expression is better represented by contact-like geometries than by isolated sources. Accordingly, SI = 0 was regarded as the most suitable parameter for Euler deconvolution in this study.

Apparent magnetic susceptibility (AMS) mapping (Fig. 16) shows high values concentrated at Nasser, Ghorabi, El-Gadidah, and El-Harrah mines, confirming that iron oxide-rich formations generate the observed magnetic highs^94,95^. Low-susceptibility zones correspond to non-magnetic sedimentary cover or altered rocks, providing lithological contrast for ore discrimination. Merging THGED and MGTHG into RGB composites successfully differentiated deep regional lineaments from shallow secondary features, offering a multidimensional subsurface perspective. Combining modern edge detection with upward continuation significantly enhances the resolution, reliability, and geological relevance of magnetic interpretation for mineral exploration^27^.

Continuous Energy Texture Gradient Amplitude and Potential Area (CET-GA, CET-PA) analyses refined structural complexity mapping by identifying circular magnetic patterns consistent with porphyritic intrusions, which are aligned with the main fault sets (Figs. 17 and 18). These intrusions, located at major lineament intersections, represent magmatic feeder systems or hydrothermal centers that exploited pre-existing structural weaknesses, serving as conduits for mineralizing fluids. Zones of lineament convergence and high orientation entropy (Fig. 17) spatially coincide with known mining sites and high-susceptibility anomalies^77^, indicating that iron-ore localization concentrates where NE–SW and NW–SE structures intersect. CET-derived structural complexity mapping confirms that faults, contacts, and dyke margins act as conduits for hydrothermal fluids, controlling iron ore concentration^77^. Porphyritic intrusions and basaltic bodies in the region generate secondary fracture systems, creating fluid pathways that enhance mineral deposition^16,93^, highlighting the shared tectonic and magmatic influence on iron mineralization across the broader region. Fault systems that control the Bahariya groundwater aquifers^87^ likely have a similar influence on ore deposition and fluid migration.

The present aeromagnetic interpretation was compared with the main previous structural, geological, and geophysical studies of the El-Bahariya area^2–24,29,30,43,77,89–94,96^. The comparison indicates that the dominant NE–SW and NW–SE magnetic trends extracted from the RTP, UC, THGED, and MGTHG maps are in good agreement with the main tectonic directions reported in these previous studies, confirming that the present aeromagnetic interpretation is consistent with the structural framework of the Bahariya area. In addition, the distribution of Euler deconvolution solutions supports earlier models of structurally controlled basement configuration. Most solutions indicate shallow basement conditions across large parts of the area, whereas deeper solutions are concentrated near major lineament intersections, suggesting deep-seated fault zones or intrusive centers that may have acted as conduits for mineralizing fluids. This pattern is compatible with the fault-controlled hydrogeological architecture proposed by^89,96^ and the tectonic controls on subsurface structure described by^77,89^. The present magnetic analysis also extends previous work by providing a clearer delineation of structurally complex zones through integrated RTP, UC, edge detection, AMS, CET-GA, and CET-PA processing. In particular, zones of lineament convergence, circular structural patterns, and high-susceptibility anomalies spatially correspond with known mining sectors and structurally disturbed areas, thereby linking the magnetic results with the regional fault-controlled framework described in earlier geological and geophysical studies. Overall, the agreement in structural orientation, basement architecture, and structurally controlled fluid pathways indicates that the present results corroborate previous models while offering a more refined image of the subsurface structural configuration relevant to ore localization.

Detailed multi-method integration: Random Forest lithological classification (Fig. 4d, OA = 91.73%, Kappa = 89.99%) delineates low-Mn, Fe-rich lithofacies (Group 1) that spatially overlap with AMS highs, UC-defined basement highs, and CET-derived fault corridors. PRISMA hyperspectral ferric iron indices (b33/b21, Fig. 7a) and ferrugination composites (Fig. 7e) show hematite and goethite absorption features (0.6, 0.9, 2.2 μm) concentrated along NE–SW and NW–SE structural trends. Spectral Angle Mapper (SAM) mineral mapping identifies 21.51 km² of hematite and 5.29 km² of limonite (Fig. 9), clustered around known mines and extending into under-explored southern sectors along the same lineament corridors. Field observations document fractures, folds, and iron-oxide-filled joints in the Naqb–Qazzun Formation at sites directly on aeromagnetic lineaments and CET boundaries (Fig. 3e, f). Geochemically, Group 1 high-grade ironstones (FeO^t^ 26–46 wt%, MnO < 0.11 wt%; Table 2) with elevated trace-element concentrations (V, W, Zn, Cr, Ni) concentrate along structural corridors (Fig. 19), indicating that hydrothermal metal input and supergene refinement were structurally focused.

The polygenetic ore-formation model is thus structurally anchored: marine hydrogenous iron precipitation (apatite-bearing, low Al₂O₃/TiO₂; Table 2) was intensified and localized by fault-controlled hydrothermal metallization (Group 1 trace-element enrichment), with subsequent supergene enrichment along the same fracture network. Paleofracture pathways remain structurally active, as evidenced by topographic expression (fault scarps, offset formations). The integrated aeromagnetic (UC/RTP/THGED/MGTHG/EUD/CET), remote sensing (RF, PRISMA, SAM), geochemical (Table 2; Fig. 19), and field evidence (Fig. 3e, f) defines a practical exploration template: prospective zones are those where NE–SW or NW–SE faults and lineament intersections (Figs. 12 and 17) coincide with (1) high SAM hematite/limonite abundance (21.51 + 5.29 km², Fig. 9), (2) RF-classified low-Mn iron lithofacies (Fig. 4d), (3) strong PRISMA ferric-iron indices (Fig. 7), and (4) elevated AMS susceptibility (Fig. 16). Global studies confirm positive correlation between magnetic field intensity variations and iron ore mineralization^96–100^, supporting the robust geophysical framework for predictive mineral exploration. Structurally focused, spectrally and magnetically corroborated zones offer the highest potential for reserve extension in the central and southern El-Bahariya depression.

### Geochemical discrimination of economic versus non-economic iron oxides

Whole-rock geochemical analysis (Table 2) reveals a clear partitioning between economic and non-economic iron-bearing lithofacies. Low-Mn ferruginous ironstones (samples BR3–BR7) contain high total iron (FeOt = 25.99–46.05 wt%, with BR7 meeting the 47.6 wt% industrial threshold) with negligible manganese (< 0.11 wt% MnO), whereas carbonate-hosted samples (BR1, BR9) show very low iron (< 5 wt%), high manganese (MnO = 4.85–7.76 wt%), and dramatically elevated barium (Ba = up to 9935 ppm) an order of magnitude higher than low-Mn ores (5–413 ppm). This Fe–Mn fractionation and the enrichment in fluid-mobile elements (V = 15–846 ppm, Zn = 85–311 ppm, W up to 387 ppm) are inconsistent with simple lateritic weathering but instead reflect chemogenic precipitation from mixed hydrogenous and hydrothermal sources. Our data align with previous studies^33,35,42^ that document Eocene Bahariya ores as having both marine hydrogenous signatures (preserved oolitic fabrics and negative Ce anomalies) and hydrothermal fingerprints (positive Eu/Y anomalies, barite, and elevated fluid-mobile element contents). The high Ba and Zn, coupled with the aeromagnetic and Sentinel-1 radar lineament patterns (NE–SW and NW–SE structures; Figs. 10 and 17) identified in this study, indicate that fault-controlled hydrothermal venting onto the seafloor supplied additional metals and sulfate during ore deposition, creating the observed geochemical signature.

Manganese enrichment in basal, carbonate-hosted horizons reflects post-depositional supergene processes that superimpose themselves on the primary ore. Baioumy et al^33^. showed that high-Mn ores occur at the stratigraphic base beneath low-Mn economic ore, containing botryoidal Mn-oxide minerals (pyrolusite, cryptomelane, bixbyite) and microbial textures and mineral assemblages consistent with precipitation by descending oxidizing fluids. Dabous^101^ documented the presence of jarosite and alunite in Bahariya ore, indicative of acidic weathering, and showed that uranium isotope ratios (²³⁴U/²³⁸U) reflect interaction with Nubian aquifer water. Importantly, the Nubian sandstone aquifer is depleted in mobile elements (Zn, Mn, Ba, U) relative to fresh sandstone, demonstrating that these elements were selectively leached and reprecipitated in the overlying Bahariya ore, a classic signature of supergene concentration. This supergene overprint acted on an earlier depositional ore protore, concentrating Mn in secondary oxides within basal pockets and oxidizing iron to hematite/goethite/jarosite assemblages in the upper, mineable horizons.

The three-group geochemical classification, derived from whole-rock major and trace element analysis (Fig. 19; Table 2), provides a quantitative criterion for discriminating between economic and non-economic lithofacies. Group 1 samples (BR3–BR7: iron-rich low-Mn ironstones with FeO^t^ = 26–46 wt%, MnO < 0.11 wt%) represent ore-grade material comparable to El-Gedida mine production (47.6 wt% Fe threshold). Group 2 samples (BR1, BR8, BR9: carbonate-hosted high-Mn phases with MnO = 4.85–7.76 wt%, FeOt < 5 wt%) represent economically non-viable, supergene-enriched basal lenses. Group 3 (barren siliceous rocks: SiO₂ ~88 wt%, FeOt < 5 wt%) represents dilutant quartz-cemented lithologies. This geochemical tripartition reflects fundamentally distinct ore-formation processes: Group 1 shows evidence of chemogenic precipitation (low Al₂O₃, TiO₂; elevated P₂O₅ indicating apatite) with hydrothermal metal input (high V, W, Zn, Cr, Ni), consistent with a polygenetic model (hydrogenous foundation → hydrothermal metallization → supergene refinement). Crucially, the Group 1 geochemical signature translates directly to distinctive spectral properties: elevated FeOt correlates with strong hematite/goethite absorption in the PRISMA ferric iron index (b33/b21; Fig. 7a), while low MnO and low Al₂O₃ avoid spectral confusion from Mn-oxide or phyllosilicate interference.

We propose a four-stage polygenic model that reconciles these observations.

#### Stage 1 (Hydrogenous)

Initial Fe oxyhydroxide precipitation from anoxic Eocene seawater, recorded in oolitic grain morphologies and negative Ce anomalies.

#### Stage 2 (Hydrothermal)

Coeval venting of Ba–Zn–V–W–rich hydrothermal brines along faults (identified based on aeromagnetic and radar datasets) replaced host carbonates and precipitated barite.

#### Stage 3 (Redox fractionation)

During early diagenesis, dissolved Mn²⁺ accumulated at redox boundaries within carbonate interbeds, with microbial catalysis producing fine-grained Mn-oxide assemblages at the base of the section.

#### Stage 4 (Supergene enrichment)

Post-Eocene arid weathering and infiltration of fresh Nubian aquifer water leached and reprecipitated Mn oxides (pyrolusite, cryptomelane) in basal lenses and oxidized iron to mature hematite/goethite/jarosite assemblages. This fourfold evolution explains the sharp geochemical and mineralogical contrasts observed, the inverted Mn stratigraphy, and the creation of the low-impurity, economically viable ore zones.

### Prospective zoning for reserve extension

**Fig. 20:**
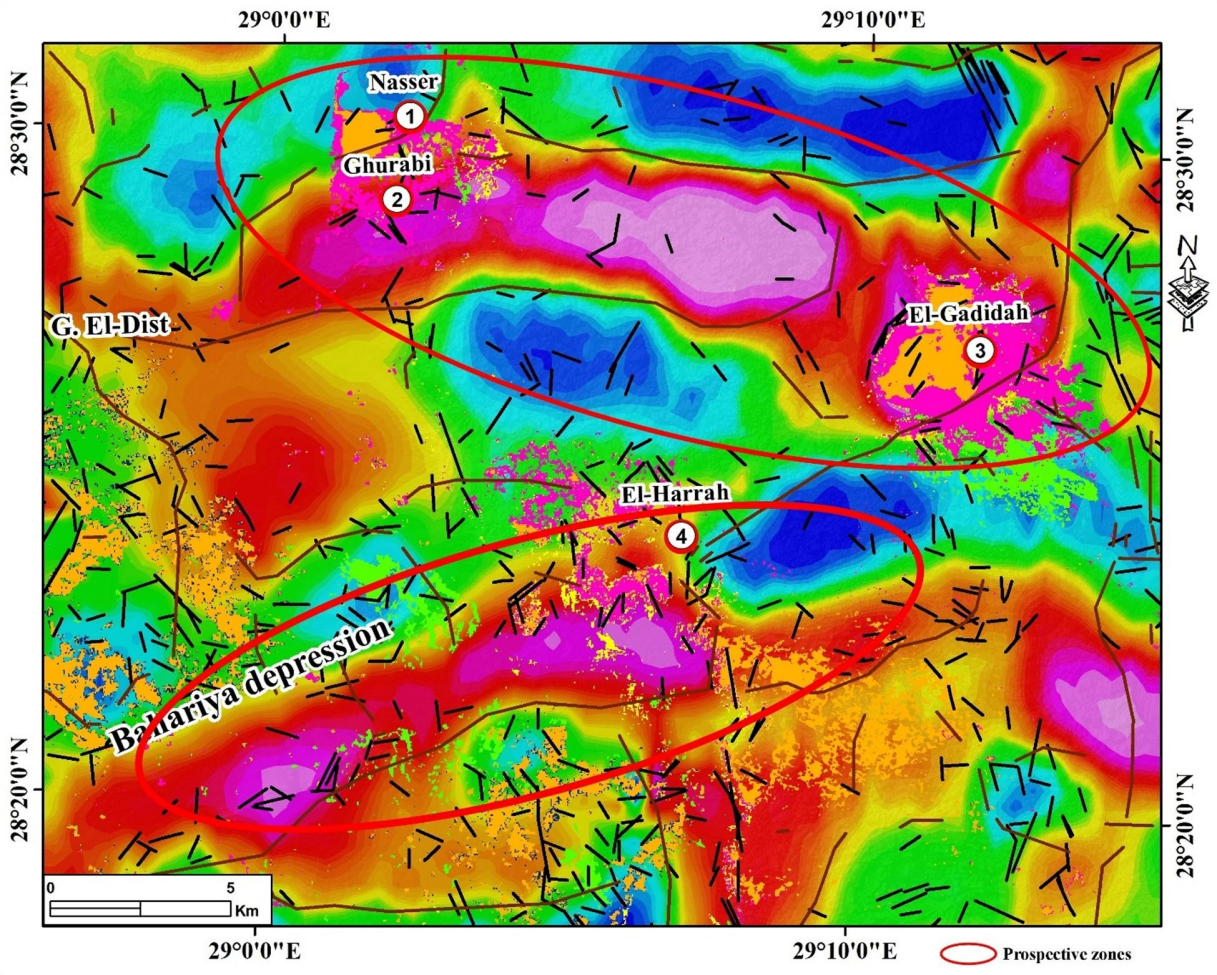
Prospective map for reserve extension exploration in El-Bahariya derived from PRISMA mineral indices and aeromagnetic structural mapping. The figure was created by ArcGIS Desktop 10.8. (https://www.esri.com/enus/arcgis/products/arcgis- 148 desktop/overview/).

PRISMA mineral indices and aeromagnetic fault mapping delineate a high-confidence prospective map for reserve extension (Fig. 20). Ferric oxide anomalies (magenta) concentrate at Nasser, Ghorabi, El-Gadidah, and El-Harrah mines and extend 5–15 km along NE–SW and NW–SE fault corridors into unmapped territory. Ferrugination halos (orange) indicate supergene enrichment. Two red-outlined prospective zones indicate high-priority targets where spectral signatures (magenta + orange), structural alignment (fault intersections), and multi-method validation (RF low-Mn lithofacies, aeromagnetic susceptibility, geochemical analysis of economic ore criteria; Table 2) converge. Three exploration corridors are identified: (A) Northern corridor from Nasser-Ghorabi extending southwest, (B) Central-eastern from El-Gadidah extending > 15 km, and (C) Southern from El-Harrah extending > 10 km. The prospective map reduces the exploration footprint to drill-ready targets. Drill sites should target high-intensity magenta pixels at fault intersections within spectral anomaly corridors at depths < 2 km (EUD estimates. This integrated spectral-structural-geochemical framework demonstrates that systematic, multi-method mineral exploration combining hyperspectral remote sensing, machine learning classification, aeromagnetic structural analysis, and ground-truth geochemistry provides a replicable and high-confidence approach to reserve extension targeting applicable to similar iron oxide deposits throughout the Arabian-Nubian Shield and other Precambrian cratonic regions worldwide.

## Conclusions

1. This study successfully mapped iron ore deposits in the Bahariya Oasis using advanced remote sensing and high-resolution aeromagnetic datasets.
2. Image processing techniques like band ratioing, PCA, MNF, PPI, and SAM effectively highlighted rock units rich in iron oxides and iron silicates. Spectral mineral indices of hyperspectral PRISMA data enabled the clear identification and mapping of various rock types and iron-rich zones.
3. Remote sensing accurately detected hematite, limonite, and iron silicates and calculated their surface areas around existing mines. Field investigations and chemical analysis confirmed the accuracy of the remote sensing results.
4. Machine learning algorithms like SVM and RF accurately map the different lithofacies in the area, with new promising zones identified, demonstrating that the efficiency of the remote sensing approach used in this study can be applied in other areas with similar geology for iron ore exploration.
5. High-resolution aeromagnetic data revealed strong magnetic anomalies, especially in the northern and northwestern parts of the study area. Edge detection filters (THGED and MGTHG) enhanced the interpretation of magnetic data and facilitated the localization of potential mineral zones.
6. Structural analysis revealed important geological features (lineaments and contacts) that may control where iron ore is found. Many of the identified magnetic anomalies align with known mines, suggesting more areas with untapped potential.
7. Most magnetic sources are within 2 km of the surface, making them relatively easy to reach for mining.
8. Overall, combining remote sensing and aeromagnetic data is a powerful and cost-effective method for exploring iron ore deposits in Bahariya and similar regions worldwide.

## Data Availability

All the data derived from this research are presented in the enclosed figures and supplementary materials.
